# Supplementary material for: hnRNP R promotes O-GlcNAcylation of eIF4G and facilitates axonal protein synthesis
Source: Nat Commun. 2024 Aug 28;15:7430. doi: 10.1038/s41467-024-51678-y (PMC11358521; doi:10.1038/s41467-024-51678-y)
Supplement: Supplementary file 1 — Supplementary Information [file 41467_2024_51678_MOESM1_ESM.pdf]

Supplementary Information for

hnRNP R promotes O-GlcNAcylation of eIF4G and facilitates axonal protein synthesis

Abdolhossein Zare, Saeede Salehi, Jakob Bader, Cornelius Schneider, Utz Fischer, Alexander Veh, Panagiota Arampatzi, Matthias Mann, Michael Briese, Michael Sendtner

This PDF file includes:

Supplementary Figures 1 to 6

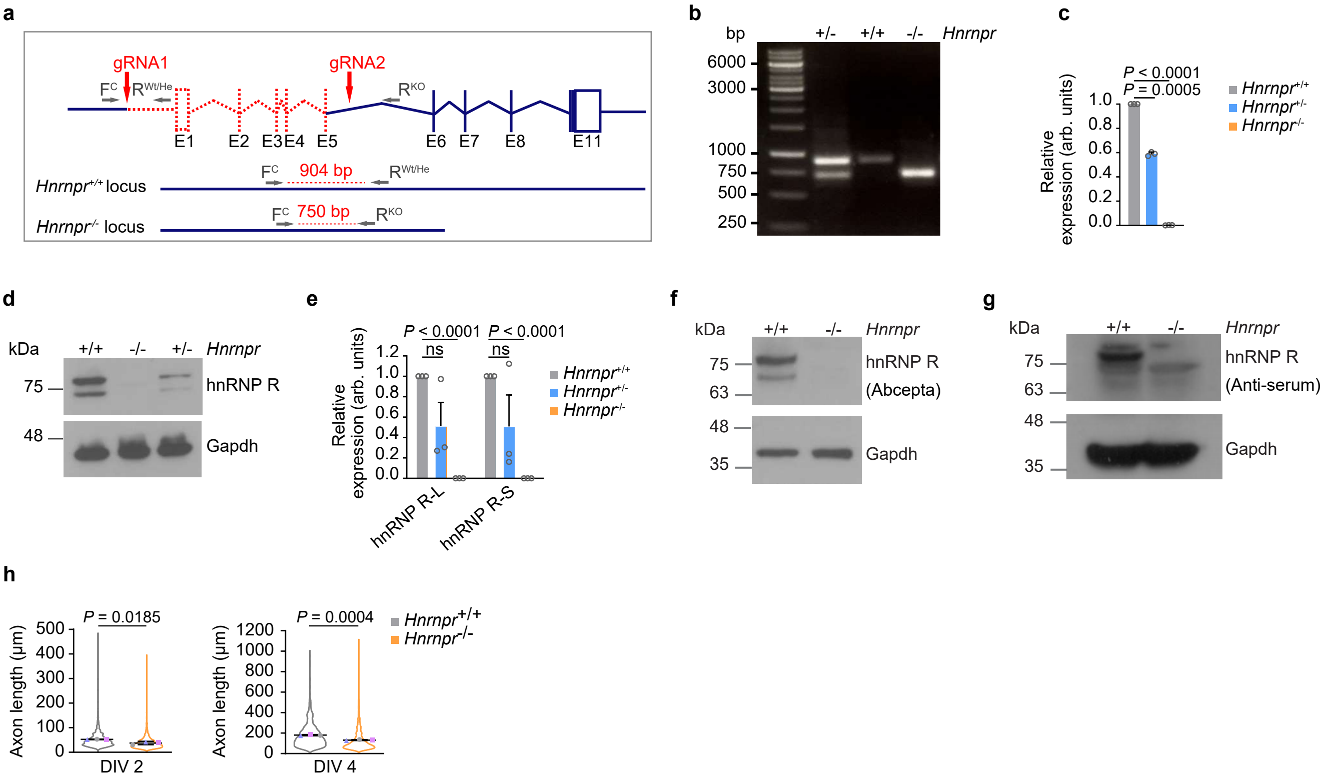

Supplementary Figure 1. hnRNP R modulates axon growth in motoneurons

a, Strategy for the generation of *Hnrnpr* knockout mice by CRISPR/Cas9 genome editing. Gray arrows represent genotyping primers. Red arrows denote the targeting sites of gRNAs. **b**, PCR genotyping results for *Hnrnpr* wildtype (+/+), heterozygous (+/-), and homozygous (-/-) deletion mutant mice. **c**, Quantification of *Hnrnpr* mRNA levels in brain lysates from E13 mice by qPCR analysis. *Gapdh* mRNA was used for normalization. Statistical analysis was performed using a two-tailed one-sample t-test. Data are mean ± standard deviation (s.d.) of three biological replicates. **d**, Immunoblot analysis of hnRNP R protein levels in E13 brain lysates using an antibody against the C-terminal domain of hnRNP R. Gapdh was used as loading control. **e**, Quantification of immunoblots as shown in (**d**). Statistical analysis was performed using a two-tailed one-sample t-test. ns, not significant. Data are mean ± s.d. of three biological replicates. **f**,**g**, Immunoblot analysis of hnRNP R protein levels in E13 brain lysates from *Hnrnpr*$^{-/-}$ and +/+ mice using an antibody against the N-terminal domain of hnRNP R from Abcepta (**f**) or anti-serum (**g**). Gapdh was used as a loading control. The immunoblot is representative of two biological replicates. **h**, SuperPlots of axon lengths of cultured motoneurons from *Hnrnpr*$^{-/-}$ and +/+ mice at DIV 2 and 4. Statistical analysis was performed using an unpaired two-tailed Student's t-test. Data are mean ± s.d. of three biological replicates. DIV 2: *Hnrnpr*$^{+/+}$, n = 1807; *Hnrnpr*$^{-/-}$, n = 1596 motoneurons. DIV 4: *Hnrnpr*$^{+/+}$, n = 530; *Hnrnpr*$^{-/-}$, n = 1056 motoneurons. Source data are provided as a Source Data file.

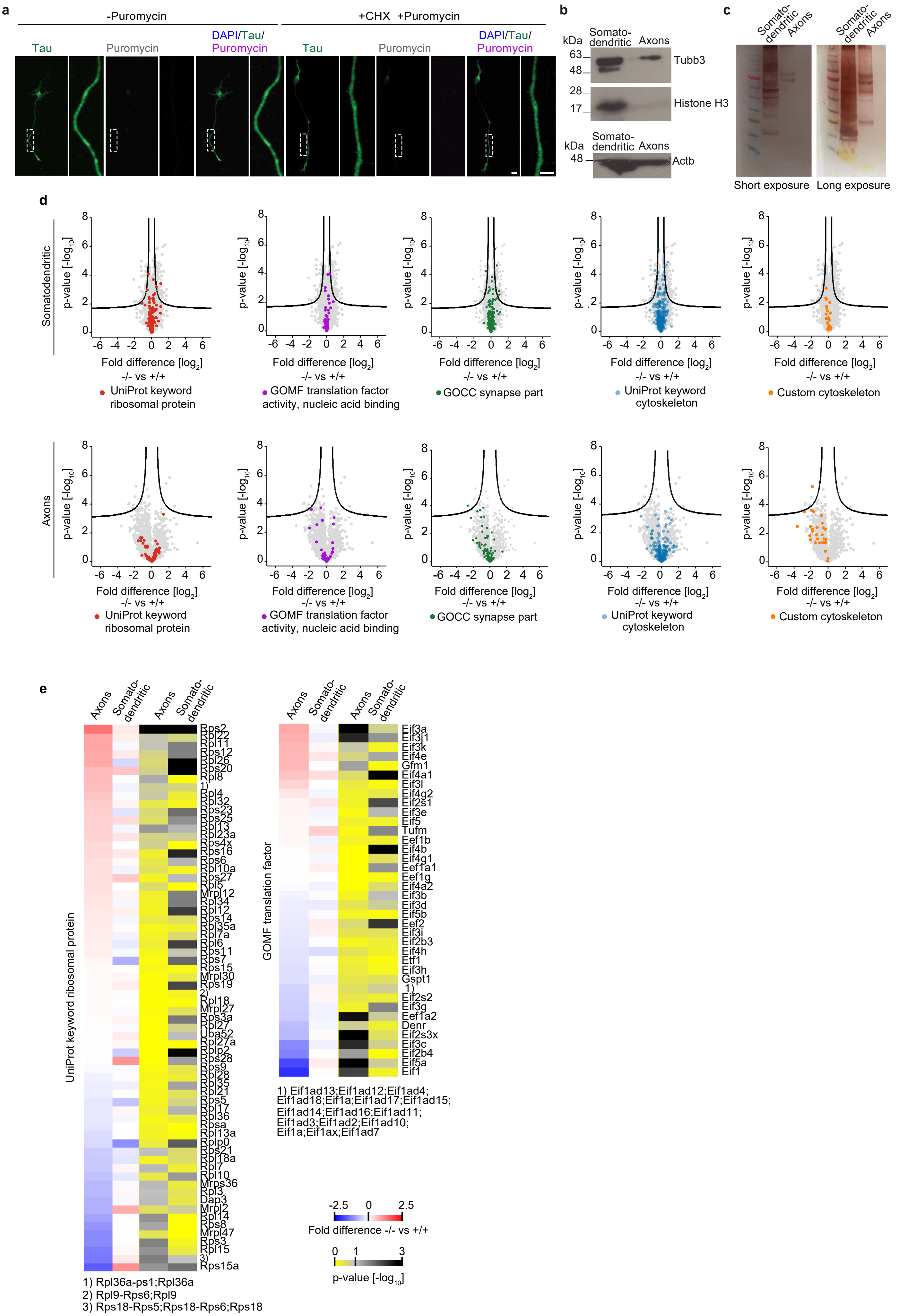
a
-Puromycin
+CHX +Puromycin
Tau
Puromycin
DAPI/Tau/Puromycin
b
kDa
Somato-dendritic
Axons
63
48
Tubb3
28
17
Histone H3
48
Actb
c
Somato-dendritic
Axons
Short exposure
Long exposure
d
Somatodendritic
Axons
p-value [-log10]
Fold difference [log2] -/- vs +/+
UniProt keyword ribosomal protein
GOMF translation factor activity, nucleic acid binding
GOCC synapse part
UniProt keyword cytoskeleton
Custom cytoskeleton
e
UniProt keyword ribosomal protein
GOMF translation factor
1) Rpl36a-ps1;Rpl36a
2) Rpl9-Rps6;Rpl9
3) Rps18-Rps5;Rps18-Rps6;Rps18
1) Eif1ad13;Eif1ad12;Eif1ad4;Eif1ad18;Eif1a;Eif1ad17;Eif1ad15;Eif1ad14;Eif1ad16;Eif1ad11;Eif1ad3;Eif1ad2;Eif1ad10;Eif1a;Eif1ax;Eif1ad7
Fold difference -/- vs +/+
p-value [-log10]

Supplementary Figure 2. Axonal proteome alterations in *Hnrnpr*$^{-/-}$ motoneurons.

a, Representative images of puromycin immunostaining of cultured motoneurons without puromycin pulse or pulsed with puromycin in the presence of cycloheximide (CHX). Scale bars, 10 µm and 5 µm (magnified areas). The images are representative of three biological replicates. **b**, Immunoblot analysis of Histone H3 as a somatodendritic marker and Actb and Tubb3 as axonal markers in somatodendritic and axonal lysate of motoneurons cultured in a microfluidic chamber. The immunoblot is representative of three biological replicates. **c**, Silver staining of somatodendritic and axonal lysate subjected to SDS-PAGE. The gel is representative of at least three biological replicates. **d**, Volcano plots highlighting differential enrichment of ribosomal proteins, translation factors, synaptic proteins and cytoskeletal components in the somatodendritic and axonal compartment of *Hnrnpr*$^{-/-}$ relative to +/+ motoneurons cultured in microfluidic chambers from four biological replicates. **e**, Heatmap of logarithmized fold changes and p-values of ribosomal proteins and translation factors. Source data are provided as a Source Data file.

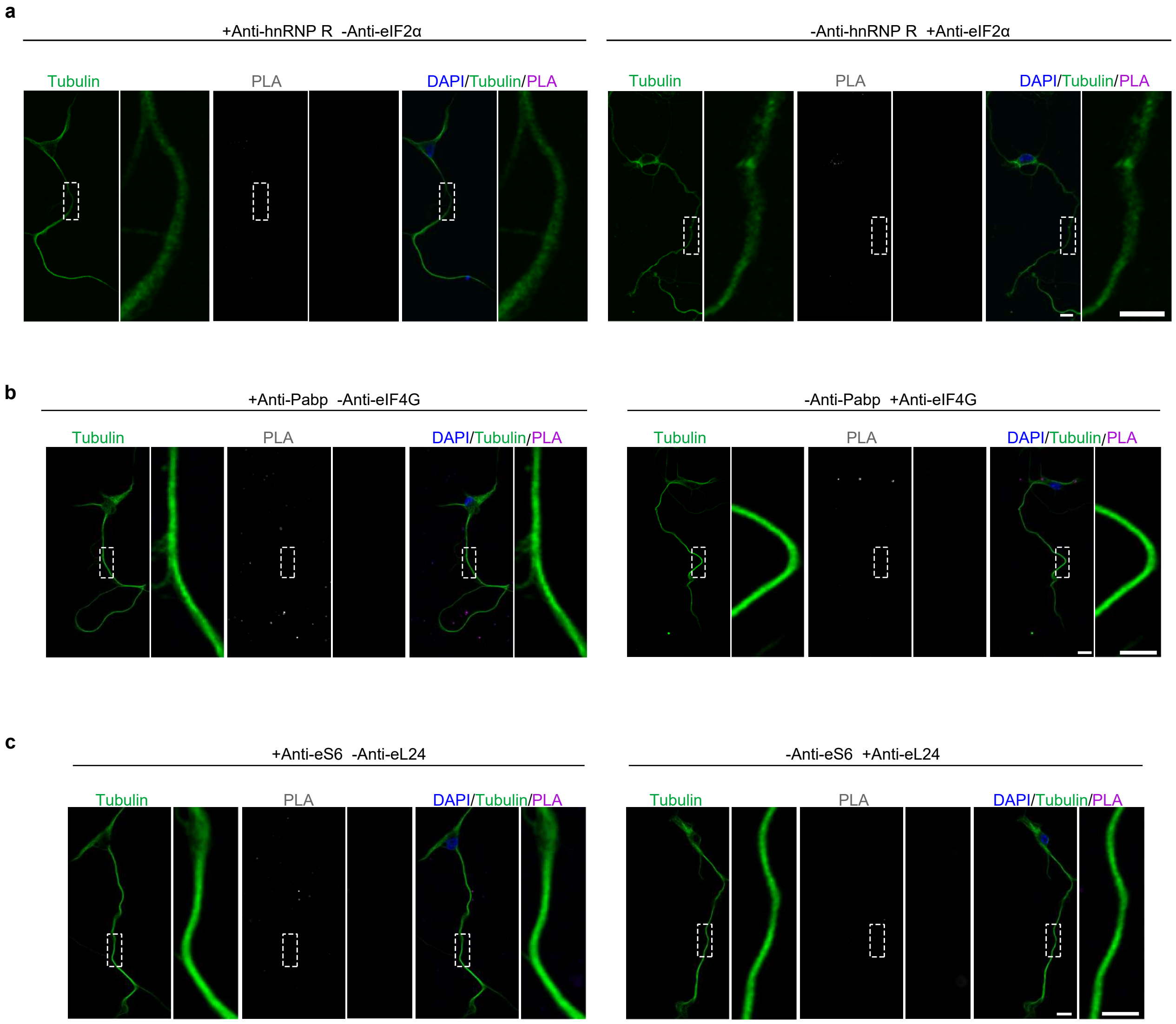

Supplementary Figure 3. Axonal translation initiation is regulated by hnRNP R in motoneurons.

a, Representative images of PLA signal in cultured DIV 6 motoneurons with either anti-eIF2α or anti-hnRNP R antibody alone as a negative control. Scale bars, 10 μm and 5 μm (magnified areas). The images are representative of two biological replicates. **b**, Representative images of PLA signal in cultured DIV 6 motoneurons with either anti-Pabp or anti-eIF4G antibody alone as a negative control. Scale bars, 10 μm and 5 μm (magnified areas). The images are representative of three biological replicates. **c**, Representative images of PLA signal in cultured DIV 6 motoneurons with either anti-eS6 or anti-eL24 antibody alone as a negative control. Scale bars, 10 μm and 5 μm (magnified areas). The images are representative of three biological replicates.

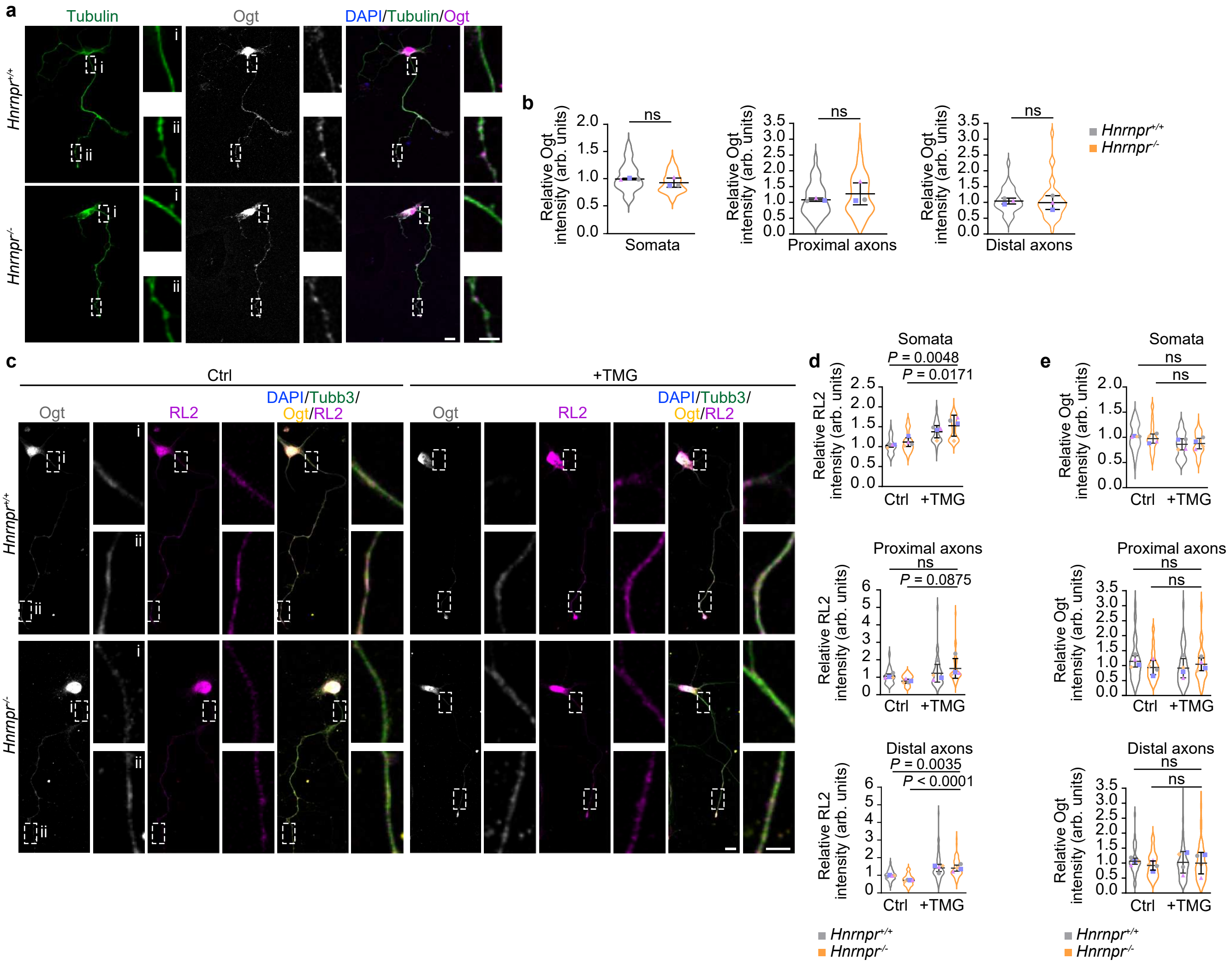

Supplementary Fig. 4 hnRNP R promotes axonal translation initiation through association with Ogt

a, Representative images of Ogt immunostaining in cultured DIV 6 *Hnrnpr*$^{-/-}$ and +/+ motoneurons. Scale bars, 10 µm, and 5 µm (magnified areas). **b**, SuperPlots of relative Ogt immunosignal intensity in somata, proximal, and distal axons of cultured *Hnrnpr*$^{-/-}$ and +/+ motoneurons. Statistical analysis was performed using an unpaired two-tailed Student's t-test. Data are mean ± s.d. of three biological replicates. *Hnrnpr*$^{+/+}$, n = 35; *Hnrnpr*$^{-/-}$, n = 39 motoneurons. **c**, Representative images of RL2 and Ogt immunostaining in cultured DIV 6 *Hnrnpr*$^{-/-}$ and +/+ motoneurons treated with DMSO (Ctrl) or thiamet G (TMG). Scale bars, 10 µm and 5 µm (magnified areas). **d**,**e**, SuperPlots of relative RL2 (**d**) and Ogt (**e**) immunosignal intensity in somata, proximal and distal axons of cultured *Hnrnpr*$^{-/-}$ and +/+ motoneurons treated with DMSO or TMG immunostained. Statistical analysis was performed using a two-way ANOVA with Tukey's multiple comparisons test. Data are mean ± s.d. of three biological replicates. Ctrl: *Hnrnpr*$^{+/+}$, n = 42; *Hnrnpr*$^{-/-}$, n = 50 motoneurons. TMG: *Hnrnpr*$^{+/+}$, n = 45; *Hnrnpr*$^{-/-}$, n = 49 motoneurons. Source data are provided as a Source Data file.

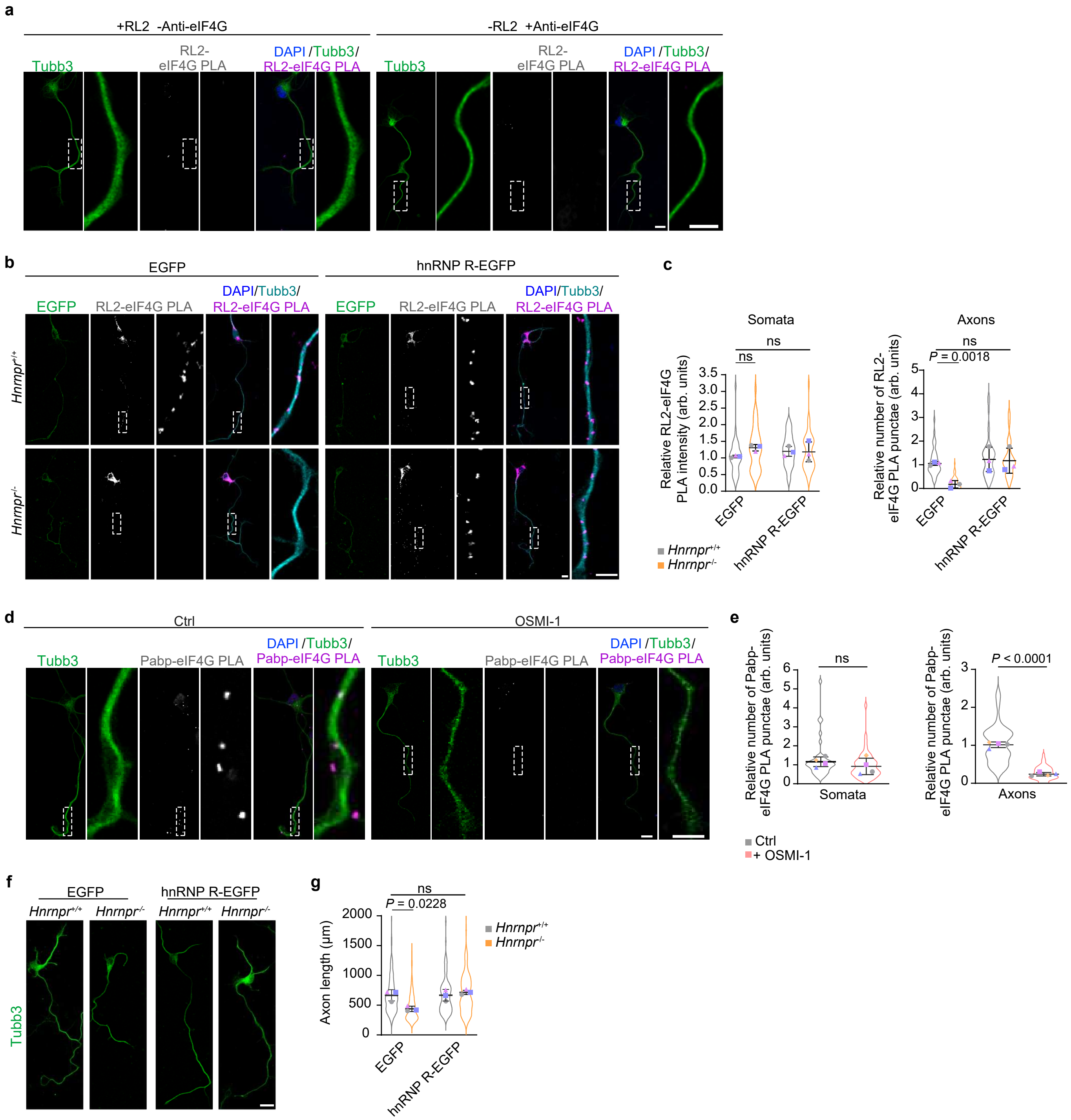

Supplementary Fig. 5 hnRNP R re-expression rescues eIF4G O-GlcNAcylation and axon growth

a, Representative images of PLA signal in cultured DIV 6 motoneurons with either RL2 or anti-eIF4G antibody alone as a negative control. Scale bars, 10 µm, and 5 µm (magnified areas). The images are representative of three biological replicates. **b**, Representative images of RL2-eIF4G PLA signal in cultured DIV 6 *Hnrnpr*$^{-/-}$ and +/+ motoneurons transduced with lentivirus expressing either EGFP or EGFP-hnRNP R. Scale bars, 10 µm and 5 µm (magnified areas). **c**, SuperPlots of the relative RL2-eIF4G PLA signal intensity in somata and number of punctae in 50 µm of axons of cultured DIV 6 *Hnrnpr*$^{-/-}$ and +/+ motoneurons transduced with lentivirus expressing either EGFP or EGFP-hnRNP R. Statistical analysis was performed using a two-way ANOVA with Tukey's multiple comparisons test. Data are mean ± s.d. of three biological replicates. EGFP: *Hnrnpr*$^{+/+}$, n = 40; *Hnrnpr*$^{-/-}$, n = 42 motoneurons. hnRNP R-EGFP: *Hnrnpr*$^{+/+}$, n = 44; *Hnrnpr*$^{-/-}$, n = 44 motoneurons. **d**, Representative images of Pabp-eIF4G PLA signal in in cultured motoneurons treated with DMSO (Ctrl) or OSMI-1. Scale bars, 10 µm and 5 µm (magnified areas). **e**, SuperPlots of the relative number of Pabp-eIF4G PLA punctae in somata and 50 µm of axons of cultured motoneurons treated with DMSO (Ctrl) or OSMI-1. Statistical analysis was performed using an unpaired two-tailed Student's t-test. Data are mean±s.d. of four

biological replicates. Ctrl: n = 42 motoneurons; OSMI-1: n = 51 motoneurons. **f**, Morphology of cultured motoneurons from *Hnrnpr*$^{-/-}$ and +/+ mice transduced with lentivirus expressing either EGFP or EGFP-hnRNP R immunostained with an antibody against tau. Scale bar, 10 µm. **g**, SuperPlots of axon lengths of cultured *Hnrnpr*$^{-/-}$ and +/+ motoneurons transduced with lentivirus expressing either EGFP or EGFP-hnRNP R. Statistical analysis was performed using a two-way ANOVA with Tukey's multiple comparisons test. Data are mean±s.d. of three biological replicates. EGFP: *Hnrnpr*$^{+/+}$, n = 103; *Hnrnpr*$^{-/-}$, n = 97 motoneurons. hnRNP R-EGFP: *Hnrnpr*$^{+/+}$, n = 98; *Hnrnpr*$^{-/-}$, n = 103 motoneurons. Source data are provided as a Source Data file.

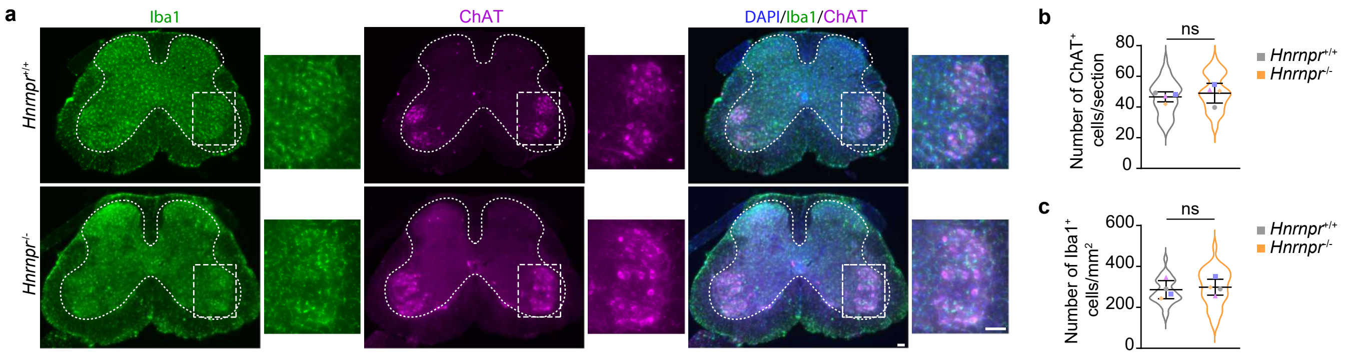

Supplementary Figure 6. Loss of hnRNP R does not affect motoneuron numbers.

a, Representative images of ChAT immunostaining of ventral horn spinal cord sections (L1-L6) from *Hnrnpr*$^{-/-}$ and +/+ mice (16 months old). Scale bars, 100 µm, and 200 µm (magnified areas). **b**,**c**, SuperPlots of number of ChAT-positive (**b**) and Iba1-positive (**c**) cells at lumbar level (L1-L6) of *Hnrnpr*$^{-/-}$ and +/+ mice. Statistical analysis was performed using an unpaired two-tailed Student's t-test. Data are mean±s.d. of four biological replicates. ChAT: *Hnrnpr*$^{+/+}$, n = 69; *Hnrnpr*$^{-/-}$, n = 45 motoneurons. Iba1: *Hnrnpr*$^{+/+}$, n = 62; *Hnrnpr*$^{-/-}$, n = 33 motoneurons. Source data are provided as a Source Data file.
