# Supplementary material for: hnRNP R promotes O-GlcNAcylation of eIF4G and facilitates axonal protein synthesis
Source: Nat Commun. 2024 Aug 28;15:7430. doi: 10.1038/s41467-024-51678-y (PMC11358521; doi:10.1038/s41467-024-51678-y)
Supplement: Supplementary file 3 — Description of Additional Supplementary Files [file 41467_2024_51678_MOESM3_ESM.pdf]

Description of Additional Supplementary Files

Supplementary Data 1. Proteomics data from the somatodendritic vs axonal compartment.

Supplementary Data 2. Proteomics data from the somatodendritic compartment of Hnrnpr-/- vs +/+ motoneurons.

Supplementary Data 3. Proteomics data from the axonal compartment of Hnrnpr-/- vs +/+ motoneurons.

Supplementary Data 4. Custom cytoskeleton annotation.

Supplementary Data 5. RNA-seq data from the somatodendritic compartment of Hnrnpr-/- vs +/+ motoneurons.

Supplementary Data 6. RNA-seq data from the axonal compartment of Hnrnpr-/- vs +/+ motoneurons.

Supplementary Data 7. Protein interactome data of hnRNP R from the somatodendritic compartment.

Supplementary Data 8. Protein interactome data of hnRNP R from the axonal compartment.

Supplementary Data 9. Protein interactome data of Ogt and for RL2 antibody from the somatodendritic compartment.

Supplementary Data 10. Protein interactome data of Ogt and for RL2 antibody from the axonal compartment.

Supplementary Data 11. List of antibodies.

Supplementary Data 12. List of qPCR primers.
