# Supplementary material for: hnRNP R promotes O-GlcNAcylation of eIF4G and facilitates axonal protein synthesis
Source: Nat Commun. 2024 Aug 28;15:7430. doi: 10.1038/s41467-024-51678-y (PMC11358521; doi:10.1038/s41467-024-51678-y)
Supplement: Supplementary file 11 — Reporting Summary [file 41467_2024_51678_MOESM11_ESM.pdf]

nature portfolio

Corresponding author(s): Michael Briese, Michael Sendtner

Last updated by author(s): Jul 26, 2024

Reporting Summary

Nature Portfolio wishes to improve the reproducibility of the work that we publish. This form provides structure for consistency and transparency in reporting. For further information on Nature Portfolio policies, see our Editorial Policies and the Editorial Policy Checklist.

Statistics

For all statistical analyses, confirm that the following items are present in the figure legend, table legend, main text, or Methods section.

n/a	Confirmed	
☐	☒	The exact sample size (*n*) for each experimental group/condition, given as a discrete number and unit of measurement
☐	☒	A statement on whether measurements were taken from distinct samples or whether the same sample was measured repeatedly
☐	☒	The statistical test(s) used AND whether they are one- or two-sided<br>*Only common tests should be described solely by name; describe more complex techniques in the Methods section.*
☒	☐	A description of all covariates tested
☒	☐	A description of any assumptions or corrections, such as tests of normality and adjustment for multiple comparisons
☐	☒	A full description of the statistical parameters including central tendency (e.g. means) or other basic estimates (e.g. regression coefficient) AND variation (e.g. standard deviation) or associated estimates of uncertainty (e.g. confidence intervals)
☐	☒	For null hypothesis testing, the test statistic (e.g. *F*, *t*, *r*) with confidence intervals, effect sizes, degrees of freedom and *P* value noted<br>*Give P values as exact values whenever suitable.*
☒	☐	For Bayesian analysis, information on the choice of priors and Markov chain Monte Carlo settings
☒	☐	For hierarchical and complex designs, identification of the appropriate level for tests and full reporting of outcomes
☐	☒	Estimates of effect sizes (e.g. Cohen's *d*, Pearson's *r*), indicating how they were calculated

Our web collection on statistics for biologists contains articles on many of the points above.

Software and code

Policy information about availability of computer code

Data collection	Xcalibur (version 3.1.66.10)
Data analysis	ImageJ (version 1.53f51), GraphPad Prism (version 9), Spectronaut (version 15.3.210906), MaxQuant (version 1.6.0.15), PERSEUS (version 1.6.15.0), bcl2fastq2 (version 2.20.0.422), Cutadapt (version 2.5), DESeq2 (version 1.24.0)

For manuscripts utilizing custom algorithms or software that are central to the research but not yet described in published literature, software must be made available to editors and reviewers. We strongly encourage code deposition in a community repository (e.g. GitHub). See the Nature Portfolio guidelines for submitting code & software for further information.

Data

Policy information about availability of data

All manuscripts must include a data availability statement. This statement should provide the following information, where applicable:

- Accession codes, unique identifiers, or web links for publicly available datasets
- A description of any restrictions on data availability
- For clinical datasets or third party data, please ensure that the statement adheres to our policy

Source data are provided with this paper. The raw mass spectrometry proteomics data have been deposited to the ProteomeXchange Consortium via the PRIDE partner repository with the dataset identifier PXD038368, PXD043851 and PXD052115. The RNA-seq data have been deposited at Gene Expression Omnibus (GEO)

under accession number GSE242027.
Previously published iCLIP dataset for hnRNP R are available at Gene Expression Omnibus (GEO) under accession code GSE77101 (https://www.ncbi.nlm.nih.gov/geo/query/acc.cgi?acc=GSE77101).
Previously published RNA-seq dataset of compartmentalized wildtype motoneurons are available at Gene Expression Omnibus (GEO) under accession code GSE66230 (https://www.ncbi.nlm.nih.gov/geo/query/acc.cgi?acc=GSE66230).

Human research participants

Policy information about studies involving human research participants and Sex and Gender in Research.

Reporting on sex and gender	N/A
Population characteristics	N/A
Recruitment	N/A
Ethics oversight	N/A

Note that full information on the approval of the study protocol must also be provided in the manuscript.

Field-specific reporting

Please select the one below that is the best fit for your research. If you are not sure, read the appropriate sections before making your selection.

☒ Life sciences ☐ Behavioural & social sciences ☐ Ecological, evolutionary & environmental sciences

For a reference copy of the document with all sections, see nature.com/documents/nr-reporting-summary-flat.pdf

Life sciences study design

All studies must disclose on these points even when the disclosure is negative.

Sample size	Most experiments were carried out independently at least in triplicates. No calculations were done to predetermine sample size. Sample size was chosen based on auther's expriences and established standards for primary neurons and tissues from animal models and cell lines.
Data exclusions	No data were excluded from the analysis.
Replication	To ensure reproducibility, all experiments except sucrose gradient fractionation were performed at least in triplicates using similar conditions and reagents. For sucrose gradient fractionation of motoneurons, two replicates were done due to the large number of cells required for the procedure. Replication was successful in all experiments.
Randomization	All experiments were carried out with primary neurons and tissues from mice with defined genetic backgrounds, and NSC-34 cells. For all experiments, samples were allocated randomly, and control and treatment conditions were derived from the same batch of cells.
Blinding	Samples for different experimental conditions were processed simultaneously such that blinding was not necessary.

Reporting for specific materials, systems and methods

We require information from authors about some types of materials, experimental systems and methods used in many studies. Here, indicate whether each material, system or method listed is relevant to your study. If you are not sure if a list item applies to your research, read the appropriate section before selecting a response.

Materials & experimental systems

n/a	Involved in the study	
☐	☒	Antibodies
☐	☒	Eukaryotic cell lines
☒	☐	Palaeontology and archaeology
☐	☒	Animals and other organisms
☒	☐	Clinical data
☒	☐	Dual use research of concern

Methods

n/a	Involved in the study	
☒	☐	ChIP-seq
☒	☐	Flow cytometry
☒	☐	MRI-based neuroimaging

Antibodies

Antibodies used

Primary antibodies:
1. Chicken polyclonal anti-Neurofilament H(NF-H), EMD Millipore, Cat# AB5539, RRID: AB_11212161.
2. Guinea pig monoclonal anti-Iba1, Synaptic Systems, Cat# 234 308, RRID: AB_2924932.
3. Guinea pig polyclonal anti-Synaptophysin, Synaptic Systems, Cat# 101004, RRID: AB_1210382.
4. Rabbit Polyclonal anti-hnRNP R, Abcam, Cat# ab30930, PRID: AB_2295539.
5. Mouse monoclonal anti-OGT, Proteintech, Cat# 66823-1-Ig, RRID: AB_2882166.
6. Mouse polyclonal anti-RPL24, Thermo Fisher Scientific, Cat# PA5-30157, RRID: AB_2547631.
7. Mouse monoclonal anti-O-Linked N-Acetylglucosamine antibody (RL2), Abcam, Cat# ab2739, RRID: AB_303264.
8. Mouse monoclonal anti-PABP, Santa Cruz Biotechnology, Cat# sc-32318, RRID: AB_628097.
9. Rabbit monoclonal anti-Eif2α, Cell Signaling Technology, Cat# 5324, RRID: AB_10692650.
10. Mouse monoclonal anti-α-Tubulin, Sigma-Aldrich, Cat# T5168, RRID: AB_477579.
11. Mouse monoclonal anti-GAPDH, EMD Millipore, Cat# CB1001, RRID: AB_2107426.
12. Mouse monoclonal anti-Puromycin, Sigma-Aldrich, Cat# MABE343, RRID: AB_2566826.
13. Mouse monoclonal anti-p75NTR, Flinders University.
14. Rabbit polyclonal anti-Tau, Sigma-Aldrich, Cat# T6402, RRID: AB_261728.
15. Mouse monoclonal anti-RPS6 (C.896.4), Thermo Fisher Scientific, Cat# MA5-15123 RRID: AB_10999800.
16. Rabbit polyclonal anti-hnRNP R, Abcepta, Cat# AP17239a, RRID: AB_11136203.
17. Mouse monoclonal anti-Ribosomal Protein S5, Santa Cruz Biotechnology, Cat# sc-390935, RRID: AB_2713966.
18. Mouse monoclonal anti-Ribosomal RNA (Y10b), Santa Cruz Biotechnology, Cat# sc-33678, RRID: AB_628226.
19. Goat Polyclonal anti-Choline Acetyltransferase (ChAT), EMD Millipore, Cat# AB144P, RRID: AB_90661.
20. Rabbit polyclonal anti-eIF4G, Cell Signaling Technology, Cat# 2498, RRID: AB_2096025.
21. Rabbit polyclonal anti-eEF2, Cell Signaling Technology, Cat# 2332, RRID: AB_10693546.
22. Rabbit polyclonal anti-Histone-H3, Abcam, Cat# ab1791, PRID: AB_302613.
23. Rabbit polyclonal anti-Macf1, Abcam, Cat# ab117418, RRID: AB_10898474.
24. Rabbit polyclonal anti-OGT, Proteintech, Cat# 11576-2-AP, RRID: AB_2156943.
25. Rabbit monoclonal anti-eIF4A(C32B4), Cell Signaling Technology, Cat# 2013, RRID:AB_2097363.
26. Rabbit polyclonal anti-hnRNP R, Sigma-Aldrich, Cat# HPA026092, RRID: AB_1850885.
27. Mouse monoclonal anti-beta-III Tubulin (TuJ-1),R&D Systems, Cat# MAB1195, RRID: AB_357520.
28. Mouse monoclonal anti-beta Actin (AC-15), GeneTex, Cat# GTX26276, RRID:AB_367161.
29. Anti- β-Tubulin 3, REAfinity, Miltenyi Biotec, Cat# 130-131-158, RRID: AB_2928186.
30. Anti-hnRNP R anti-serum, Rossoll et al, 2002. https://doi.org/10.1093/hmg/11.1.93.
31. Rabbit IgG Control, PeproTech, Cat# 500-P00, RRID: AB_2722620.
32. Mouse IgG Control, Santa Cruz Biotechnology, Cat# sc-2025, RRID: AB_737182.

Secondary antibodies:
1. Donkey Anti-Rabbit IgG (HRP), Jackson ImmunoResearch, Cat# 711-035-152, RRID: AB_10015282
2. Mouse Anti-Rabbit IgG Antibody (HRP), Jackson ImmunoResearch, Cat# 211-032-171 RRID: AB_2339149
3. Donkey Anti-Chicken-Alexa Fluor® 488, Jackson ImmunoResearch, Cat# 703-545-155 RRID: AB_2340375
4. Donkey anti-Rabbit-Alexa Fluor 647, Thermo Fisher Scientific, Cat# A31573, RRID: AB_2536183
5. Donkey anti-Mouse-Cy3, Jackson ImmunoResearch, Cat# 715-165-150, RRID: AB_2340813
6. Donkey anti-Rabbit-Cy3, Jackson ImmunoResearch, Cat# 711-166-152, RRID: AB_2313568

Validation

Primary antibodies:
1. Chicken polyclonal anti-Neurofilament H(NF-H), EMD Millipore, Cat# AB5539, RRID: AB_11212161. Validation data provided by the supplier (https://www.merckmillipore.com/FR/fr/product/Anti-Neurofilament-H-Antibody,MM_NFAB5539).
2. Guinea pig monoclonal anti-Iba1, Synaptic Systems, Cat# 234 308, RRID: AB_2924932. Validation data provided by the supplier (https://www.sysy.com/product/234308).
3. Guinea pig polyclonal anti-Synaptophysin, Synaptic Systems, Cat# 101004, RRID: AB_1210382. Validation data provided by the supplier (https://www.sysy.com/product/101004).
4. Rabbit Polyclonal anti-hnRNP R, Abcam, Cat# ab30930, PRID: AB_2295539. Validation data provided by the supplier (https://www.abcam.com/hnRNP-R-antibody-ab30930.html), validated in our lab by shRNA knock down and knock out.
5. Mouse monoclonal anti-OGT, Proteintech, Cat# 66823-1-Ig, RRID: AB_2882166. Validation data provided by the supplier
6. Mouse polyclonal anti-RPL24, Thermo Fisher Scientific, Cat# PA5-30157, RRID: AB_2547631. Validation data provided by the supplier (https://www.thermofisher.com/antibody/product/RPL24-Antibody-Polyclonal/PA5-30157).
7. Mouse monoclonal anti-O-Linked N-Acetylglucosamine antibody (RL2), Abcam, Cat# ab2739, RRID: AB_303264. Validation data provided by the supplier (https://www.abcam.com/products/primary-antibodies/o-linked-n-acetylglucosamine-antibodyrl2-

ab2739.html).
8. Mouse monoclonal anti-PABP, Santa Cruz Biotechnology, Cat# sc-32318, RRID: AB_628097.
Validation data provided by the supplier (https://www.scbt.com/p/pabp-antibody-10e10).
9. Rabbit monoclonal anti-Eif2α, Cell Signaling Technology, Cat# 5324, RRID: AB_10692650.
Validation data provided by the supplier (https://www.cellsignal.com/products/primaryantibodies/eif2a-d7d3-xp-rabbit-mab/5324).
10. Mouse monoclonal anti-α-Tubulin, Sigma-Aldrich, Cat# T5168, RRID: AB_477579.
Validation data provided by the supplier (https://www.sigmaaldrich.com/DE/de/product/sigma/t5168).
11. Mouse monoclonal anti-GAPDH, EMD Millipore, Cat# CB1001, RRID: AB_2107426.
Validation data provided by the supplier (https://www.merckmillipore.com/DE/de/product/Anti-GAPDH-Mouse-mAb-6C5,EMD_BIO-CB1001).
12. Mouse monoclonal anti-Puromycin, Sigma-Aldrich, Cat# MABE343, RRID: AB_2566826.
Validation data provided by the supplier (https://www.sigmaaldrich.com/DE/de/product/mm/mabe343).
13. Mouse monoclonal anti-p75NTR, Flinders University. Validated in our lab (Wiese et al. 2009 Nature Protocols).
14. Rabbit polyclonal anti-Tau, Sigma-Aldrich, Cat# T6402, RRID: AB_261728. Validation data provided by the supplier (https://www.sigmaaldrich.com/DE/de/product/sigma/t6402).
15. Mouse monoclonal anti-RPS6 (C.896.4), Thermo Fisher Scientific, Cat# MA5-15123 RRID: AB_10999800. Validation data provided by the supplier (https://www.thermofisher.com/antibody/product/S6-Antibody-clone-C-896-4-Monoclonal/MA5-15123).
16. Rabbit polyclonal anti-hnRNP R, Abcepta, Cat# AP17239a, RRID: AB_11136203. Validation data provided by the supplier (https://www.abcepta.com/products/AP17239a-HNRNPR-Antibody-N-term), validated in our lab by shRNA knock down and knock out.
17. Mouse monoclonal anti-Ribosomal Protein S5, Santa Cruz Biotechnology, Cat# sc-390935, RRID: AB_2713966. Validation data provided by the supplier (https://www.scbt.com/de/p/ribosomal-protein-s5-antibody-a-8).
18. Mouse monoclonal anti-Ribosomal RNA (Y10b), Santa Cruz Biotechnology, Cat# sc-33678, RRID: AB_628226. Validation data provided by the supplier (https://www.scbt.com/p/rrnaantibody-y10b/).
19. Goat Polyclonal anti-Choline Acetyltransferase (ChAT), EMD Millipore, Cat# AB144P, RRID: AB_90661. Validation data provided by the supplier (https://www.merckmillipore.com/DE/de/product/Anti-Choline-Acetyltransferase-Antibody,MM_NF-AB144P?ReferrerURL=https%3A%2F%2Fwww.bing.com%2F&bd=1).
20. Rabbit polyclonal anti-eIF4G, Cell Signaling Technology, Cat# 2498, RRID: AB_2096025.
Validation data provided by the supplier (https://www.cellsignal.com/products/primaryantibodies/eif4g-antibody/2498?_requestid=2737507).
21. Rabbit polyclonal anti-eEF2, Cell Signaling Technology, Cat# 2332, RRID: AB_10693546.
Validation data provided by the supplier (Rabbit polyclonal anti-eEF2, Cell Signaling Technology, Cat# 2332).
22. Rabbit polyclonal anti-Histone-H3, Abcam, Cat# ab1791, PRID: AB_302613. Validation data provided by the supplier (https://www.abcam.com/histone-h3-antibody-nuclear-marker-and-chipgrade-ab1791.html).
23. Rabbit polyclonal anti-Macf1, Abcam, Cat# ab117418, RRID: AB_10898474. Validation data provided by the supplier (https://www.abcam.com/products/primary-antibodies/macf1-antibodyab117418.html).
24. Rabbit polyclonal anti-OGT, Proteintech, Cat# 11576-2-AP, RRID: AB_2156943. Validation data provided by the supplier (https://www.ptglab.com/products/OGT-Antibody-11576-2-AP.htm).
25. Rabbit monoclonal anti-eIF4A(C32B4), Cell Signaling Technology, Cat# 2013, RRID: AB_2097363.Validation data provided by the supplier (https://www.cellsignal.com/products/primary-antibodies/eif4a-c32b4-rabbit-mab/2013).
26. Rabbit polyclonal anti-hnRNP R, Sigma-Aldrich, Cat# HPA026092, RRID: AB_1850885.Validation data provided by the supplier (https://www.sigmaaldrich.com/DE/en/product/sigma/hpa026092), validated in our lab by shRNA knock down and knock out in our lab.
27. Mouse monoclonal anti-beta-III Tubulin (TuJ-1),R&D Systems, Cat# MAB1195, RRID: AB_357520.Validation data provided by the supplier (https://www.rndsystems.com/products/neuron-specific-beta-iii-tubulin-antibody-tuj-1_mab1195)
28. Mouse monoclonal anti-beta Actin (AC-15), GeneTex, Cat# GTX26276, RRID:AB_367161. Validation data provided by the supplier (https://www.genetex.com/Product/Detail/beta-Actin-antibody-AC-15/GTX26276).
29. Anti- β-Tubulin 3, REAfinity, Miltenyi Biotec, Cat# 130-131-158, RRID: AB_2928186. Validation data provided by the supplier (https://www.miltenyibiotec.com/DE-en/products/b-tubulin-3-antibody-anti-human-mouse-reafinity-rea1152.html#conjugate=vio-b515:size=600-ul).
30. Anti-hnRNP R anti-serum, Validated in our lab (Rossoll et al, 2002. Human Molecular Genetics)
31. Rabbit IgG Control, PeproTech, Cat# 500-P00, RRID: AB_2722620. Validation data provided by the supplier (https://www.peprotech.com/en/normal-rabbit-igg).
32. Mouse IgG Control, Santa Cruz Biotechnology, Cat# sc-2025, RRID: AB_737182. Validation data provided by the supplier (https://www.scbt.com/p/normal-mouse-igg).

Secondary antibodies:
1. Donkey Anti-Rabbit IgG (HRP), Jackson ImmunoResearch, Cat# 711-035-152, RRID: AB_10015282. Validated in our lab by omission of primary antibody, absence of target antigen and used under similar condition for other project.
2. Mouse Anti-Rabbit IgG Antibody (HRP), Jackson ImmunoResearch, Cat# 211-032-171 RRID: AB_2339149. Validated in our lab by omission of primary antibody, absence of target antigen and used under similar condition for other project.
3. Donkey Anti-Chicken-Alexa Fluor® 488, Jackson ImmunoResearch, Cat# 703-545-155 RRID: AB_2340375. Validated in our lab by omission of primary antibody, absence of target

antigen and used under similar condition for other project.
4. Donkey anti-Rabbit-Alexa Fluor 647, Thermo Fisher Scientific, Cat# A31573, RRID: AB_2536183. Validated in our lab by omission of primary antibody, absence of target antigen and used under similar condition for other project.
5. Donkey anti-Mouse-Cy3, Jackson ImmunoResearch, Cat# 715-165-150, RRID: AB_2340813. Validated in our lab by omission of primary antibody, absence of target antigen and used under similar condition for other project.
6. Donkey anti-Rabbit-Cy3, Jackson ImmunoResearch, Cat# 711-166-152, RRID: AB_2313568. Validated in our lab by omission of primary antibody, absence of target antigen and used under similar condition for other project.

Eukaryotic cell lines

Policy information about cell lines and Sex and Gender in Research

Cell line source(s)	NSC-34 cells (Cedarlane, cat. no. CLU140) and and HEK293TN cells (System Biosciences, cat. no. LV900A-1)
Authentication	NSC-34 and HEK293TN cells were obtained commercially from Cedarlane and System Biosciences, respectively, and were not authenticated.
Mycoplasma contamination	NSC-34 and HEK293TN cells tested negative for mycoplasma contamination.
Commonly misidentified lines (See ICLAC register)	No commonly misidentified cell lines were used in the study.

Animals and other research organisms

Policy information about studies involving animals; ARRIVE guidelines recommended for reporting animal research, and Sex and Gender in Research

Laboratory animals	Hnrnpr+/+ and -/- mice on a C57Bl/6 background of both genders were housed in the animal facility of the Institute of Clinical Neurobiology at the University Hospital of Wuerzburg. Mice were maintained on a 12 h/12 h day/night cycle under controlled conditions at 20-22°C and 55-65% humidity with food and water in abundant supply. Breeding animals were between 6 and 20 weeks of age. Pregnancy in female mice was detected by daily plug control, and mouse embryos were isolated at E13 for generation of primary motoneuron cultures. Tissues were obtained from mice at defined ages as indicated in the manuscript.
Wild animals	The study did not involve wild animals.
Reporting on sex	Sex was not considered in the study design and information on sex was not collected because embryonic mice were used for motoneuron cultures.
Field-collected samples	The study did not involve samples collected from the field.
Ethics oversight	All animal experiments were performed strictly according to the regulations on animal protection of the German federal law and the Association for Assessment and Accreditation of Laboratory Animal Care, in agreement with and under the control of the local veterinary authority.

Note that full information on the approval of the study protocol must also be provided in the manuscript.
