# Supplementary figures and images for: hnRNP R promotes O-GlcNAcylation of eIF4G and facilitates axonal protein synthesis
Source: Nat Commun. 2024 Aug 28;15:7430. doi: 10.1038/s41467-024-51678-y (PMC11358521; doi:10.1038/s41467-024-51678-y)

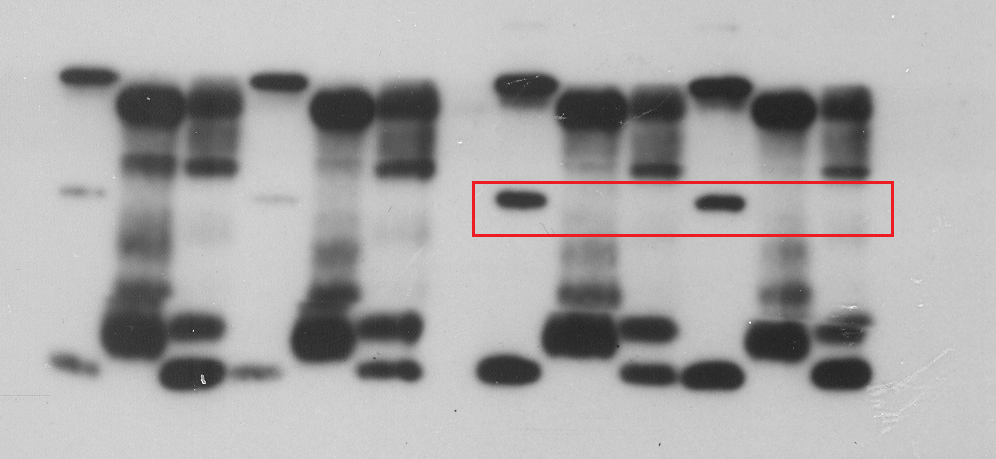

Supplement: Supplementary file 12 — Source Data [file 41467_2024_51678_MOESM12_ESM.zip › Source Data Zare et al/Fig. 5/Fig. 5a -MN-Gapdh.tif]

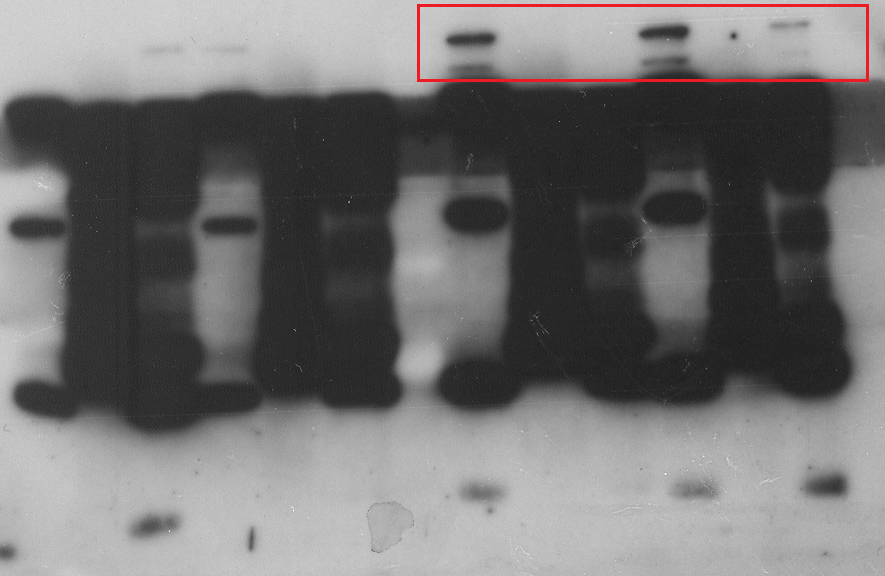

Supplement: Supplementary file 12 — Source Data [file 41467_2024_51678_MOESM12_ESM.zip › Source Data Zare et al/Fig. 5/Fig. 5a -MN-hnRNP R.tif]

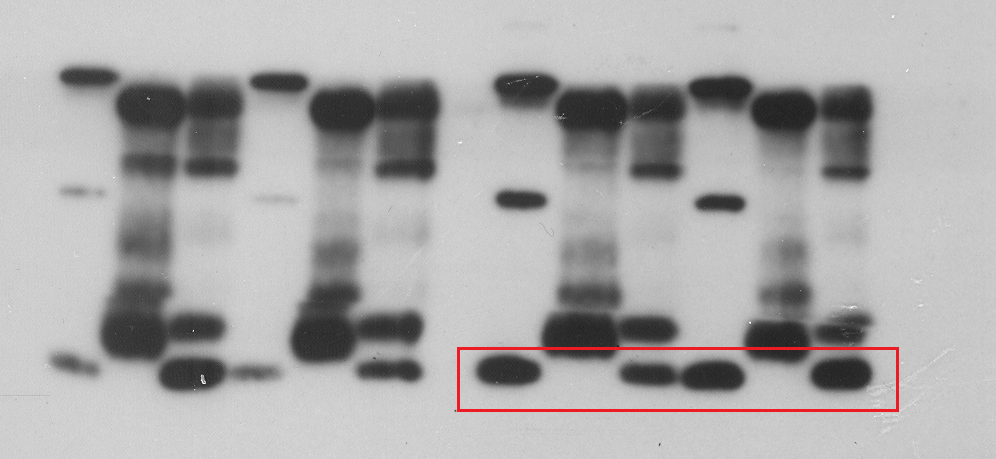

Supplement: Supplementary file 12 — Source Data [file 41467_2024_51678_MOESM12_ESM.zip › Source Data Zare et al/Fig. 5/Fig. 5a-MN-eS5.tif]

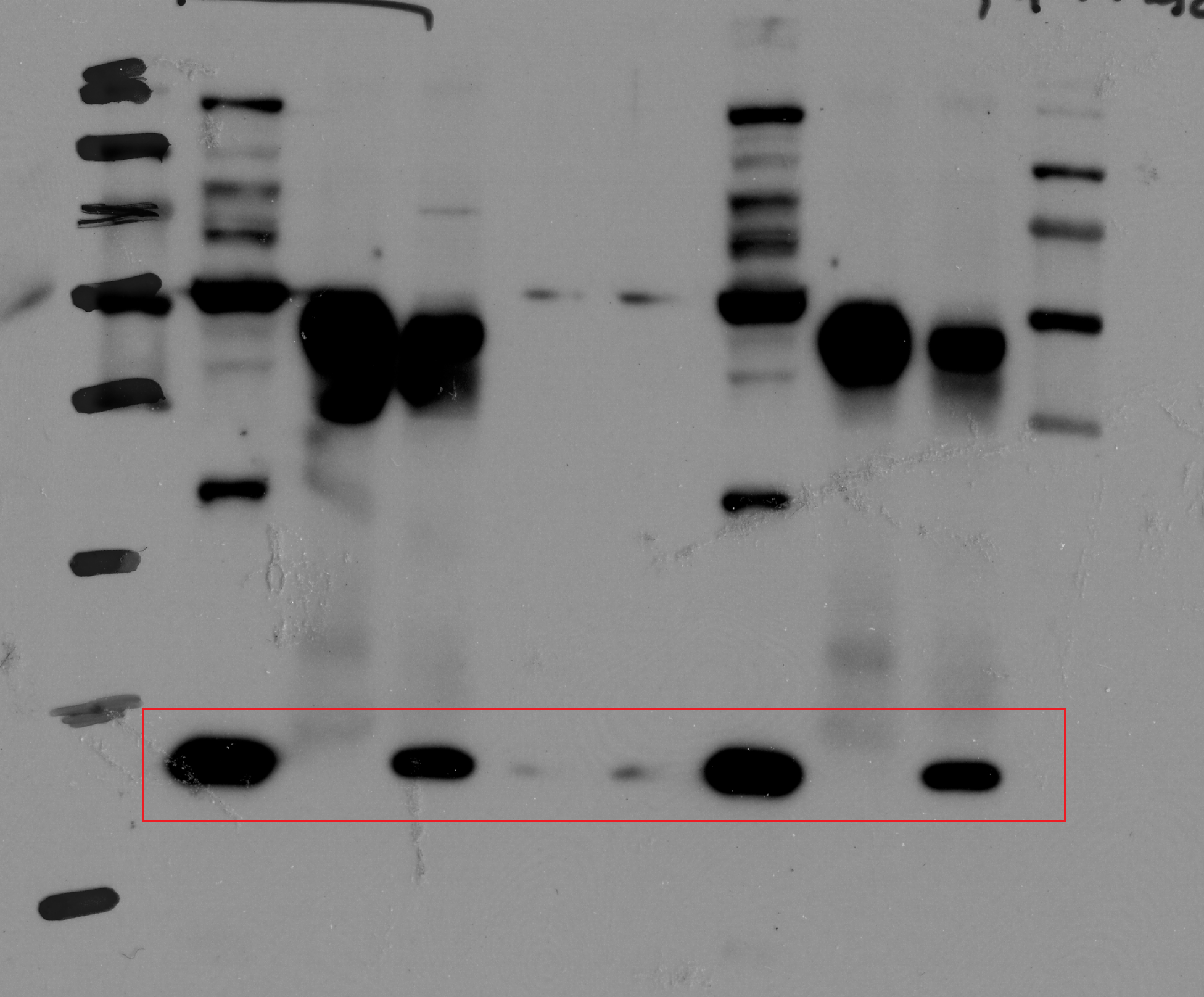

Supplement: Supplementary file 12 — Source Data [file 41467_2024_51678_MOESM12_ESM.zip › Source Data Zare et al/Fig. 5/Fig. 5a-NSC34-eS5.tif]

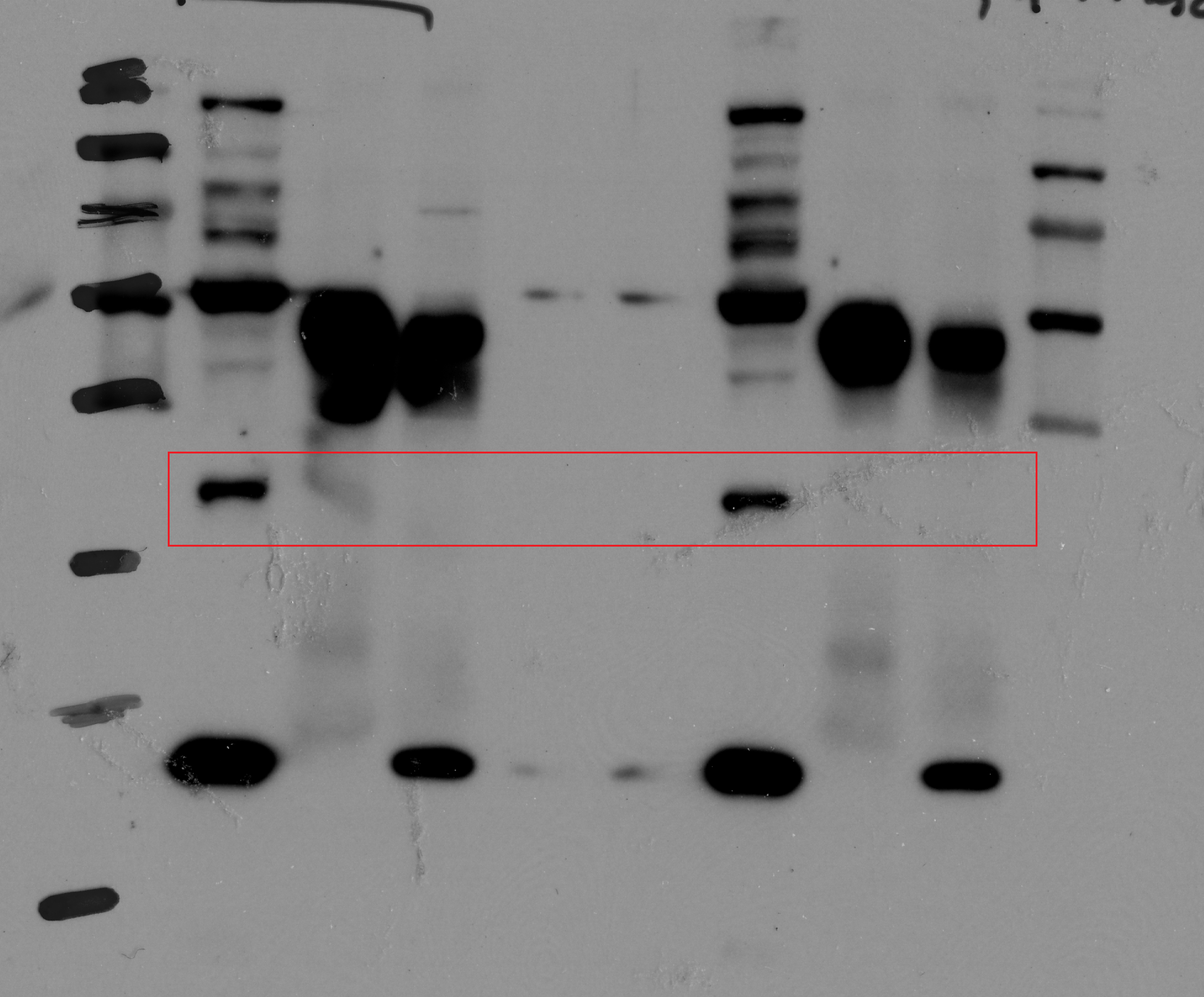

Supplement: Supplementary file 12 — Source Data [file 41467_2024_51678_MOESM12_ESM.zip › Source Data Zare et al/Fig. 5/Fig. 5a-NSC34-Gapdh.tif]

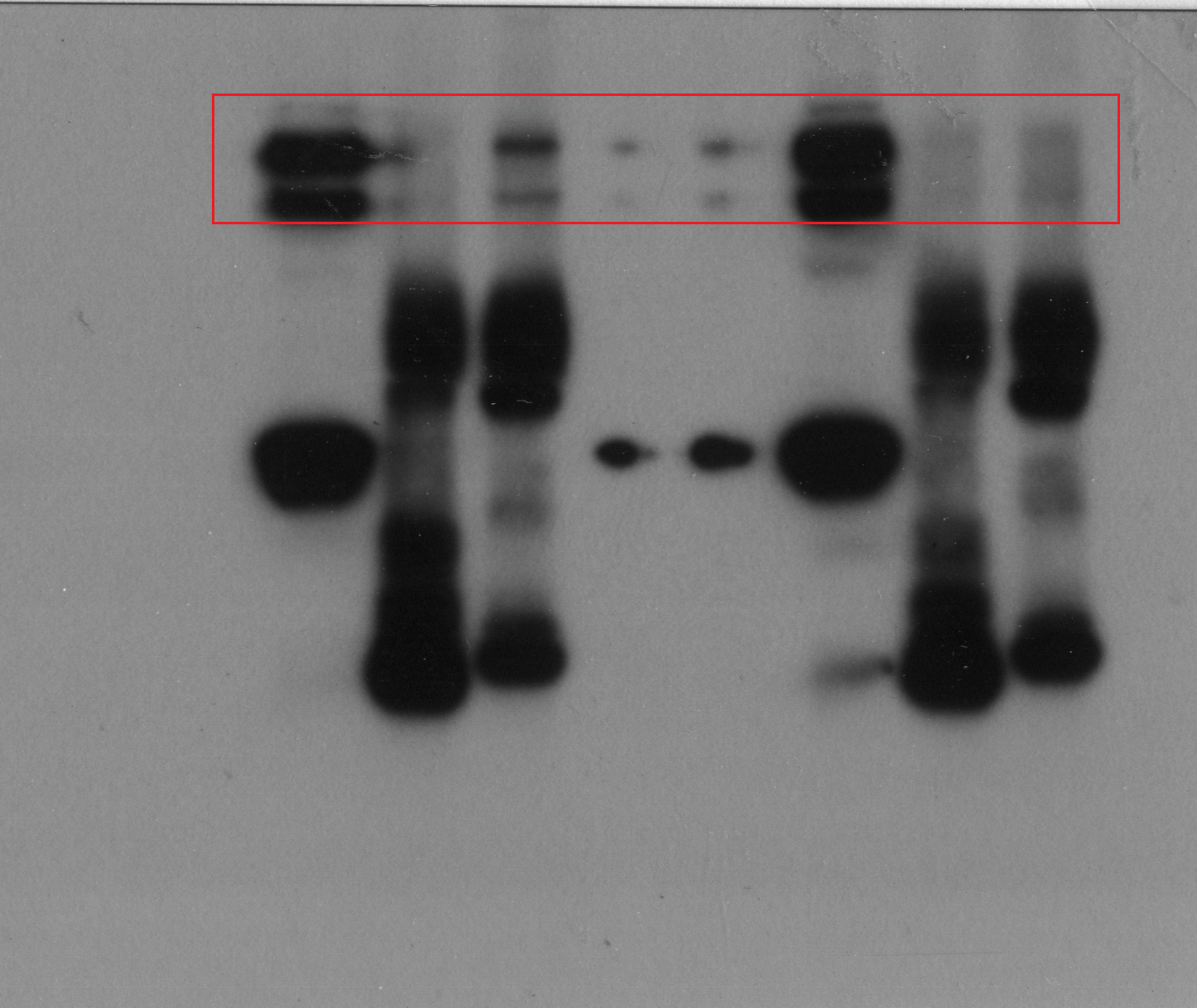

Supplement: Supplementary file 12 — Source Data [file 41467_2024_51678_MOESM12_ESM.zip › Source Data Zare et al/Fig. 5/Fig. 5a-NSC34-hnRNP R.tif]

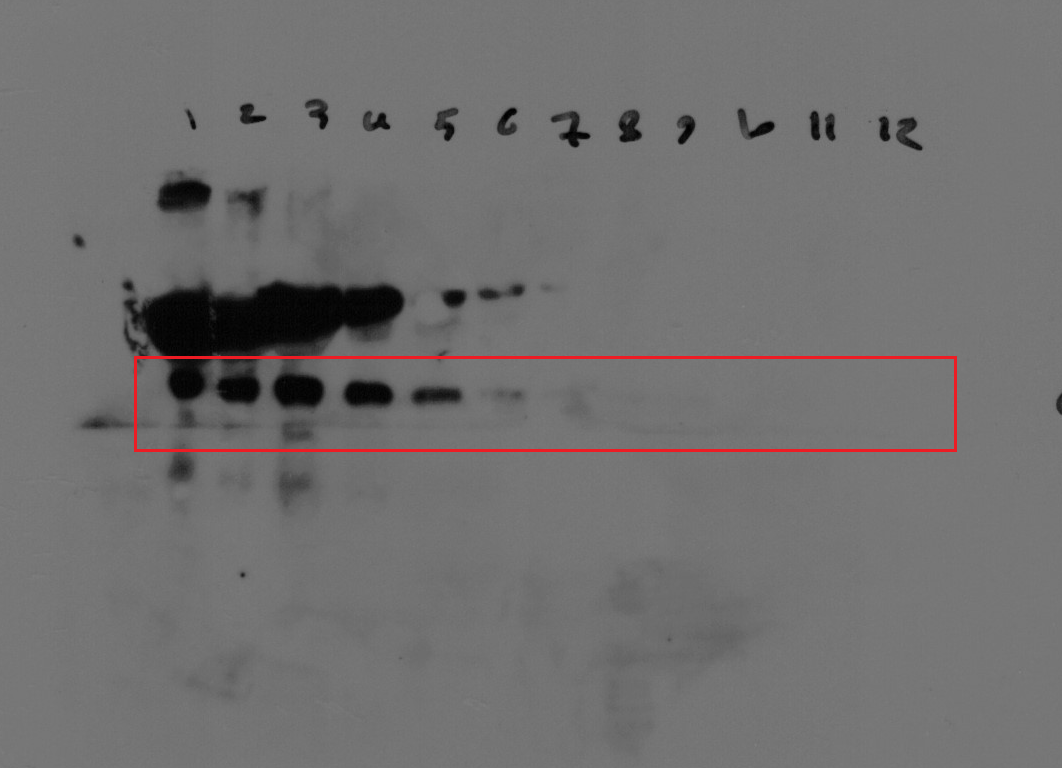

Supplement: Supplementary file 12 — Source Data [file 41467_2024_51678_MOESM12_ESM.zip › Source Data Zare et al/Fig. 5/Fig. 5e-Axons-eIF2.tif]

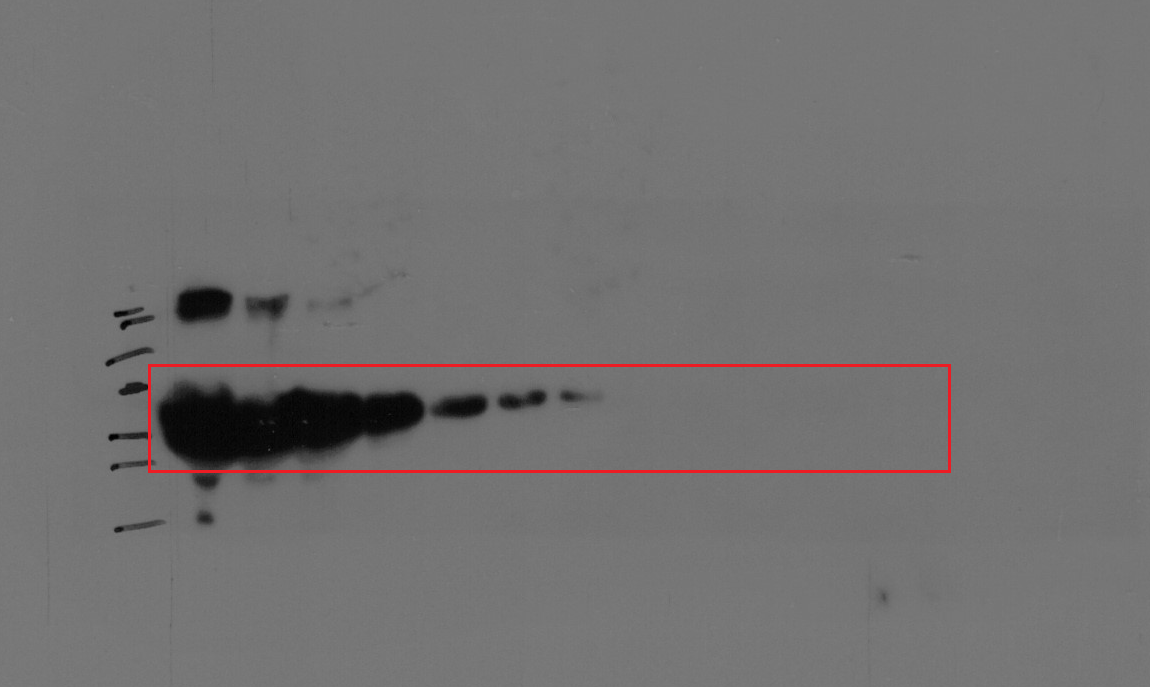

Supplement: Supplementary file 12 — Source Data [file 41467_2024_51678_MOESM12_ESM.zip › Source Data Zare et al/Fig. 5/Fig. 5e-Axons-hnRNP R.tif]

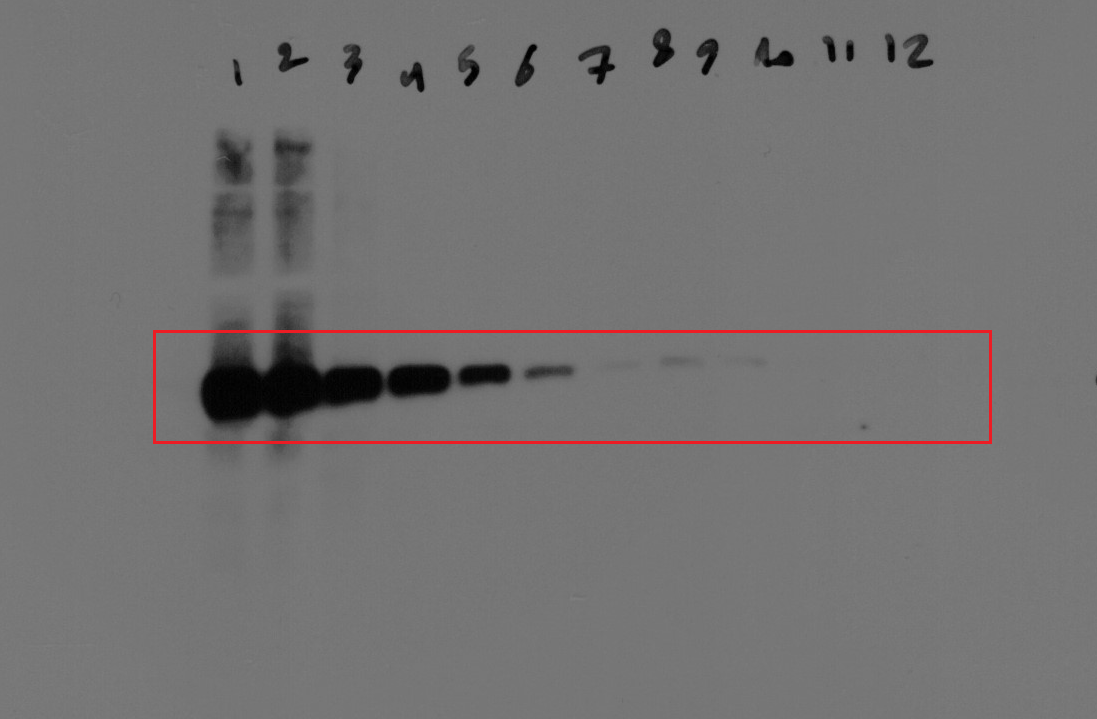

Supplement: Supplementary file 12 — Source Data [file 41467_2024_51678_MOESM12_ESM.zip › Source Data Zare et al/Fig. 5/Fig. 5e-Somata-eIF2.tif]

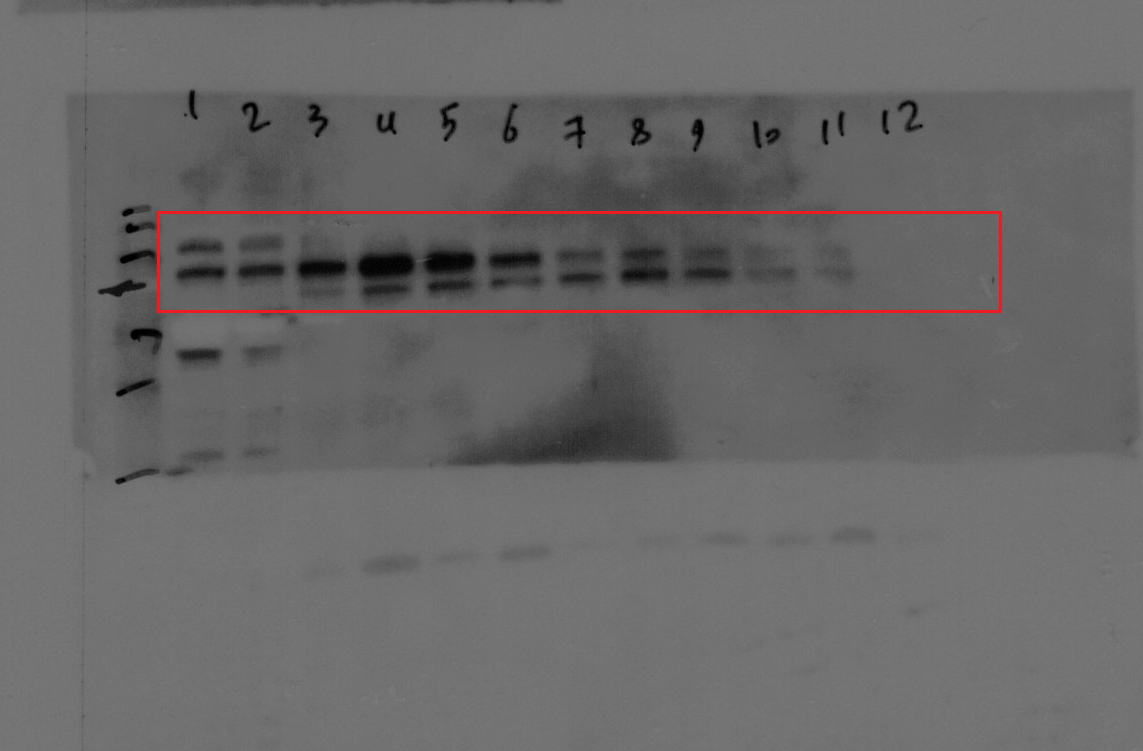

Supplement: Supplementary file 12 — Source Data [file 41467_2024_51678_MOESM12_ESM.zip › Source Data Zare et al/Fig. 5/Fig. 5e-Somata-hnRNP R.tif]

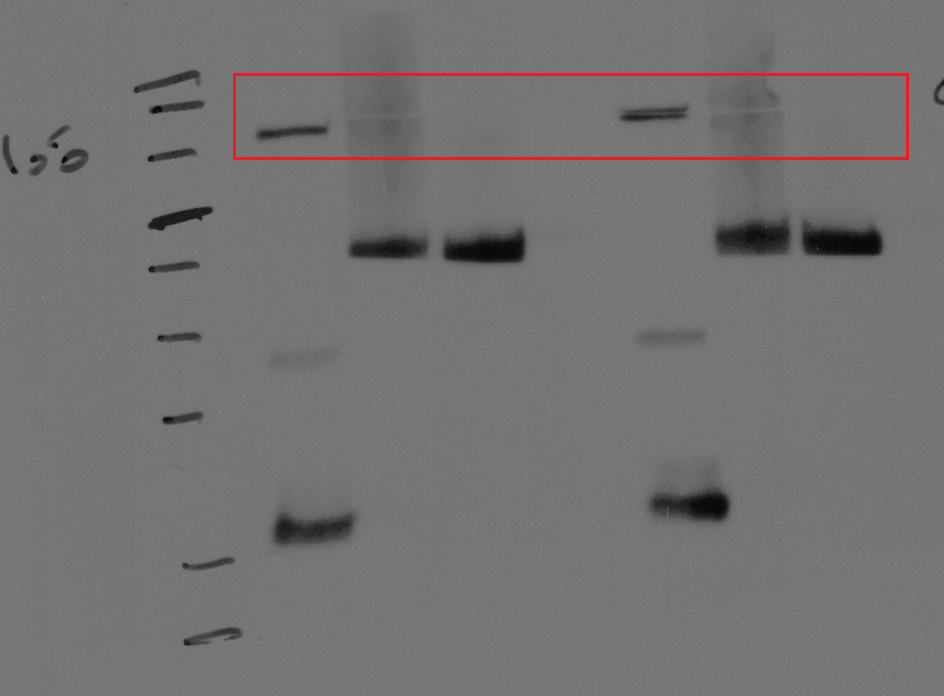

Supplement: Supplementary file 12 — Source Data [file 41467_2024_51678_MOESM12_ESM.zip › Source Data Zare et al/Fig. 5/Fig. 5g-eEF2.tif]

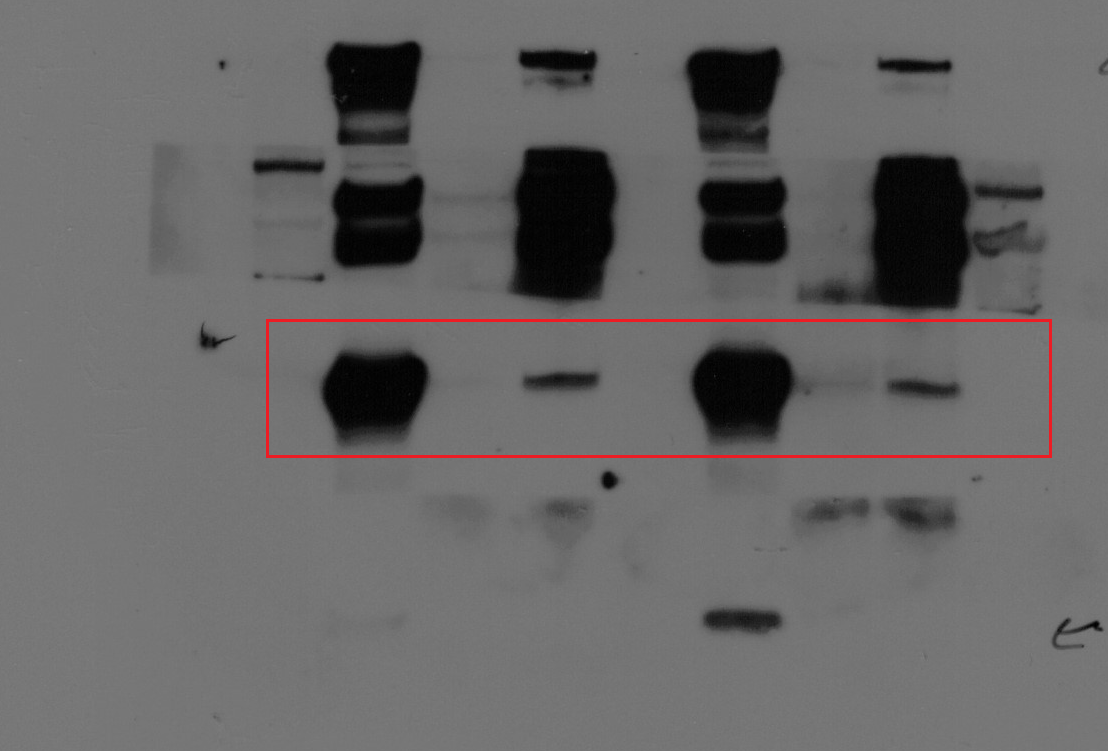

Supplement: Supplementary file 12 — Source Data [file 41467_2024_51678_MOESM12_ESM.zip › Source Data Zare et al/Fig. 5/Fig. 5g-eIF2.tif]

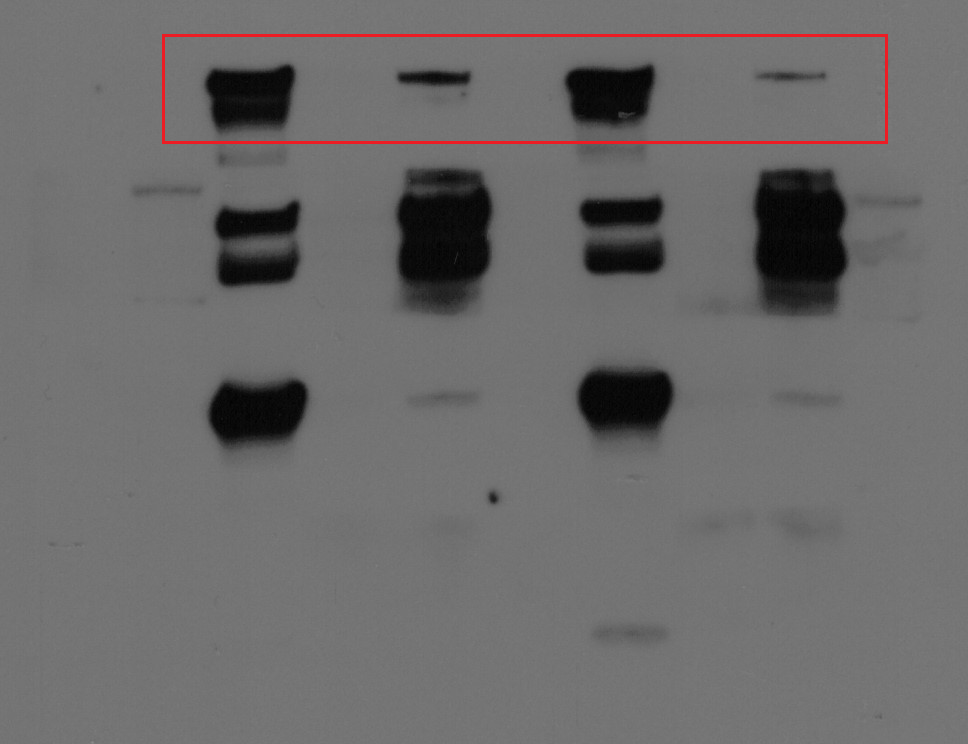

Supplement: Supplementary file 12 — Source Data [file 41467_2024_51678_MOESM12_ESM.zip › Source Data Zare et al/Fig. 5/Fig. 5g-eIF4G.tif]

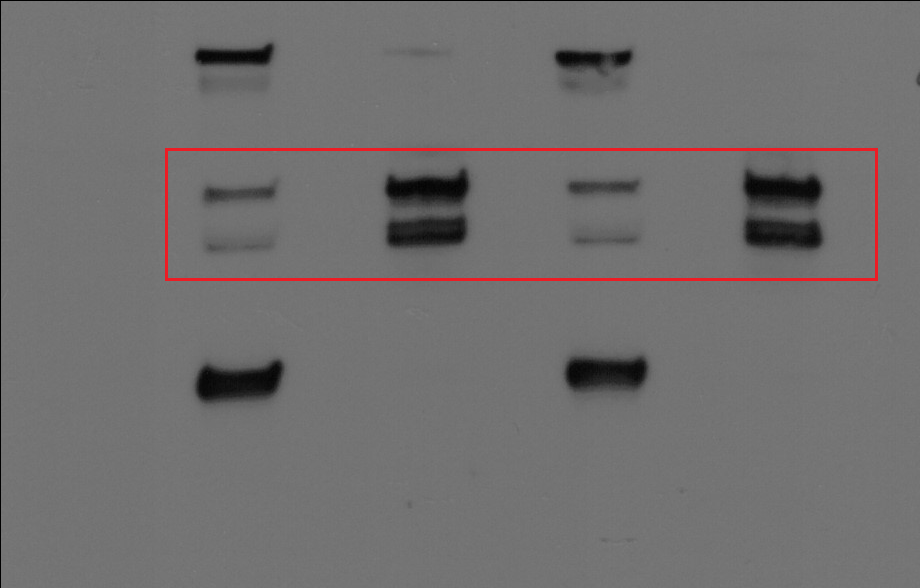

Supplement: Supplementary file 12 — Source Data [file 41467_2024_51678_MOESM12_ESM.zip › Source Data Zare et al/Fig. 5/Fig. 5g-hnRNP R.tif]

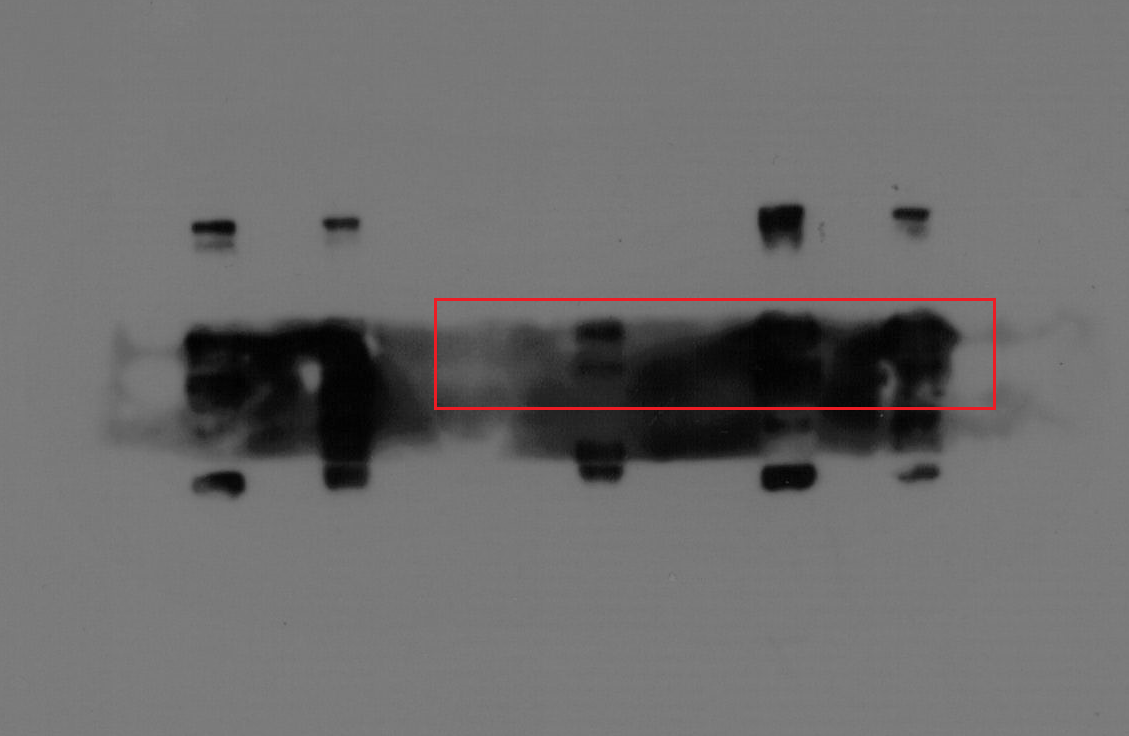

Supplement: Supplementary file 12 — Source Data [file 41467_2024_51678_MOESM12_ESM.zip › Source Data Zare et al/Fig. 7/Fig. 7f-hnRNP R-long exposure.tif]

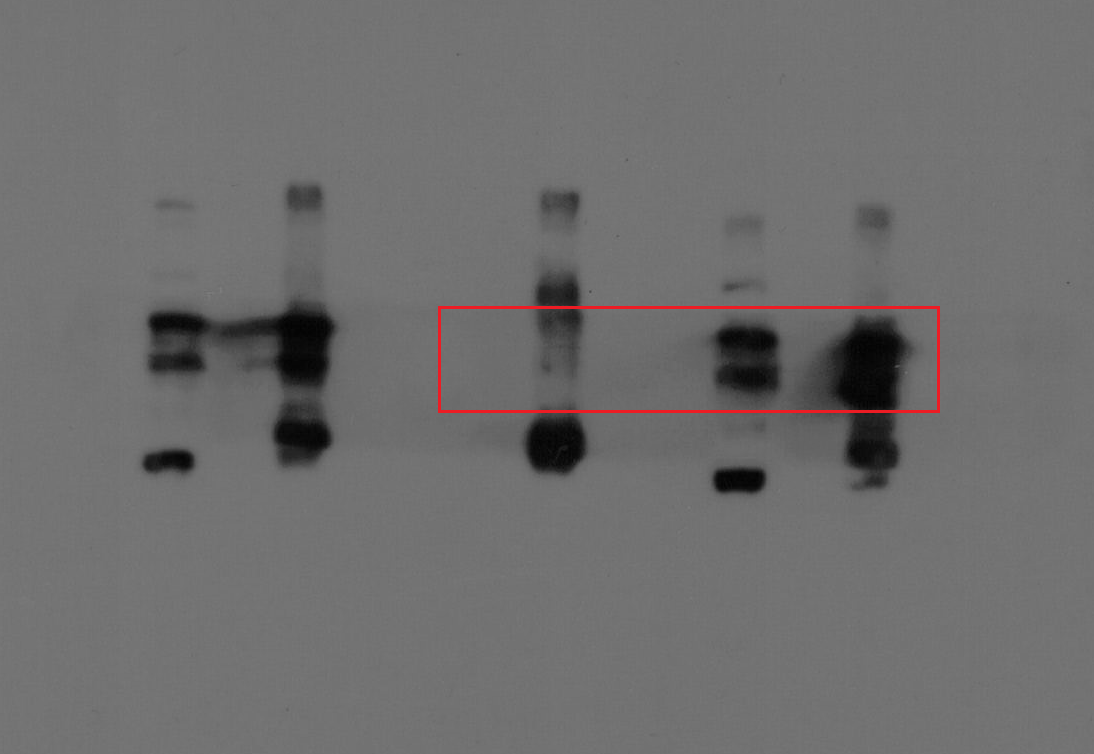

Supplement: Supplementary file 12 — Source Data [file 41467_2024_51678_MOESM12_ESM.zip › Source Data Zare et al/Fig. 7/Fig. 7f-hnRNP R-short exposure.tif]

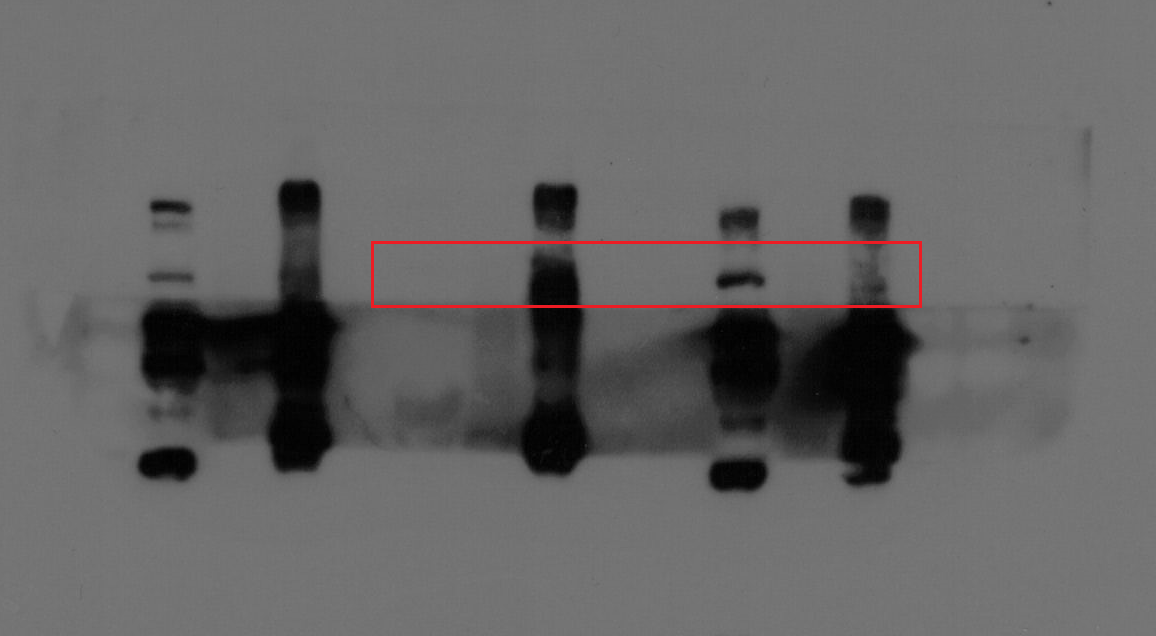

Supplement: Supplementary file 12 — Source Data [file 41467_2024_51678_MOESM12_ESM.zip › Source Data Zare et al/Fig. 7/Fig. 7f-Ogt-long exposure.tif]

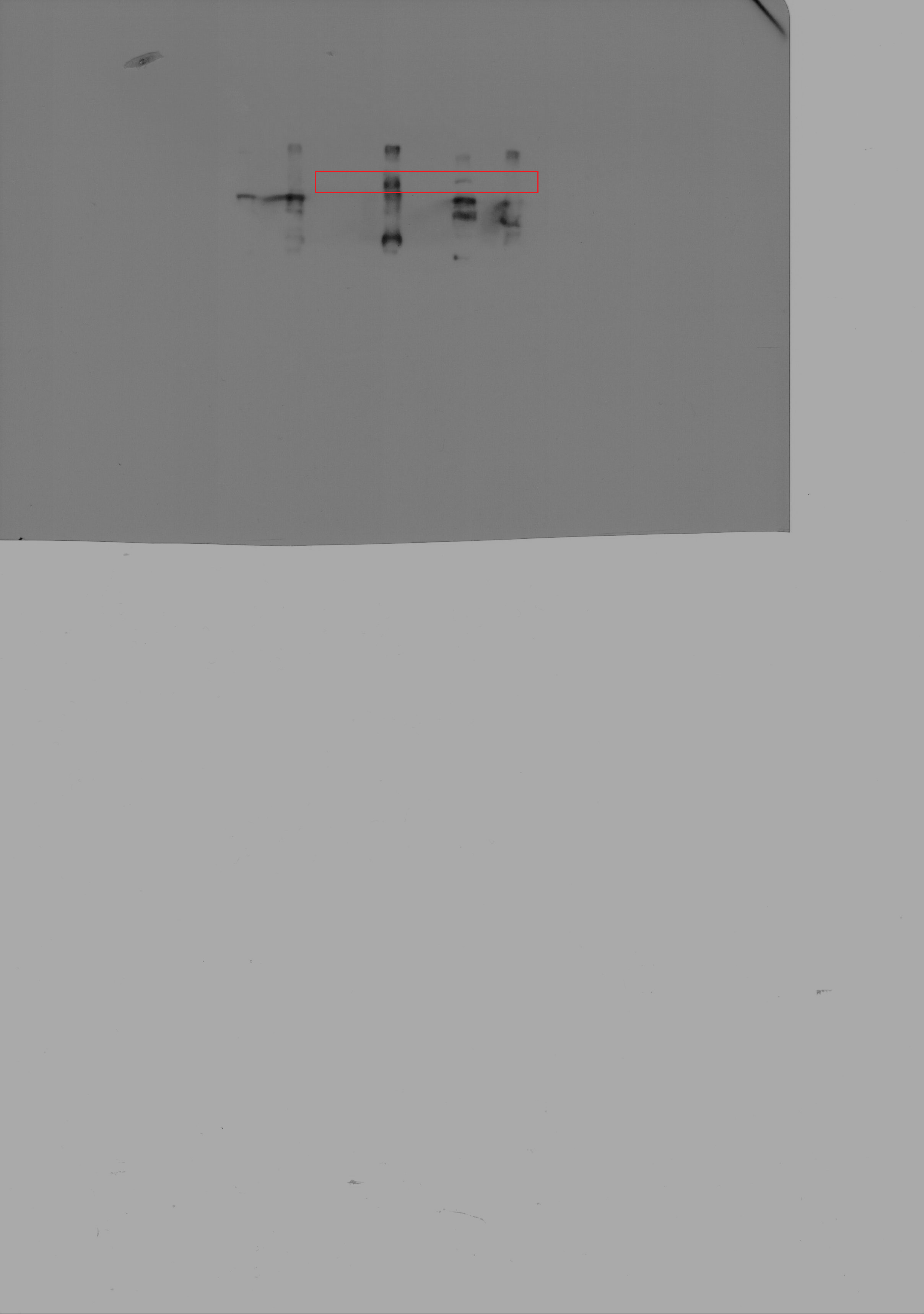

Supplement: Supplementary file 12 — Source Data [file 41467_2024_51678_MOESM12_ESM.zip › Source Data Zare et al/Fig. 7/Fig. 7f-Ogt-short exposure.tif]

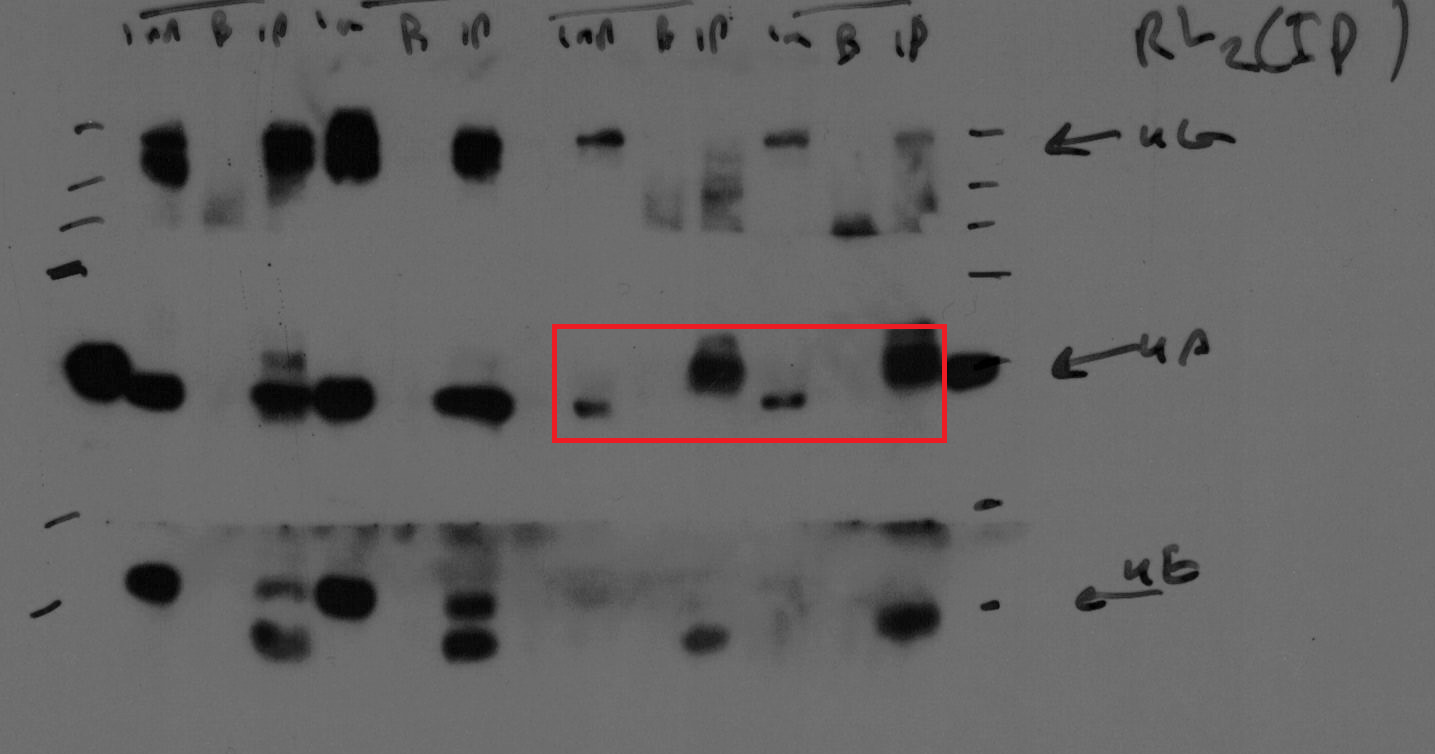

Supplement: Supplementary file 12 — Source Data [file 41467_2024_51678_MOESM12_ESM.zip › Source Data Zare et al/Fig. 8/Fig. 8d-Axons-eIF4A.tif]

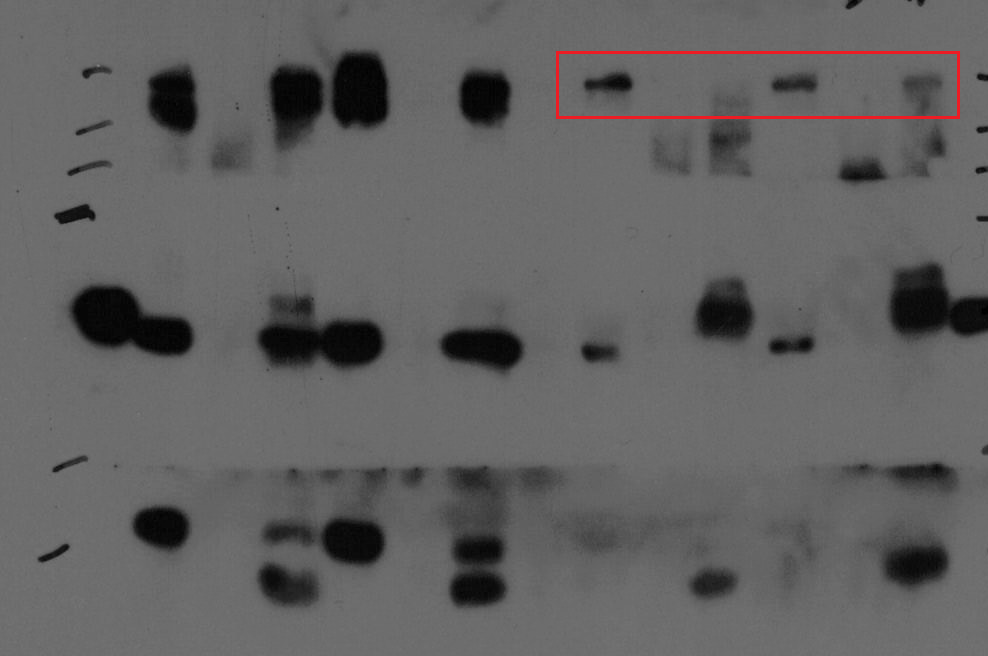

Supplement: Supplementary file 12 — Source Data [file 41467_2024_51678_MOESM12_ESM.zip › Source Data Zare et al/Fig. 8/Fig. 8d-Axons-eIF4G.tif]

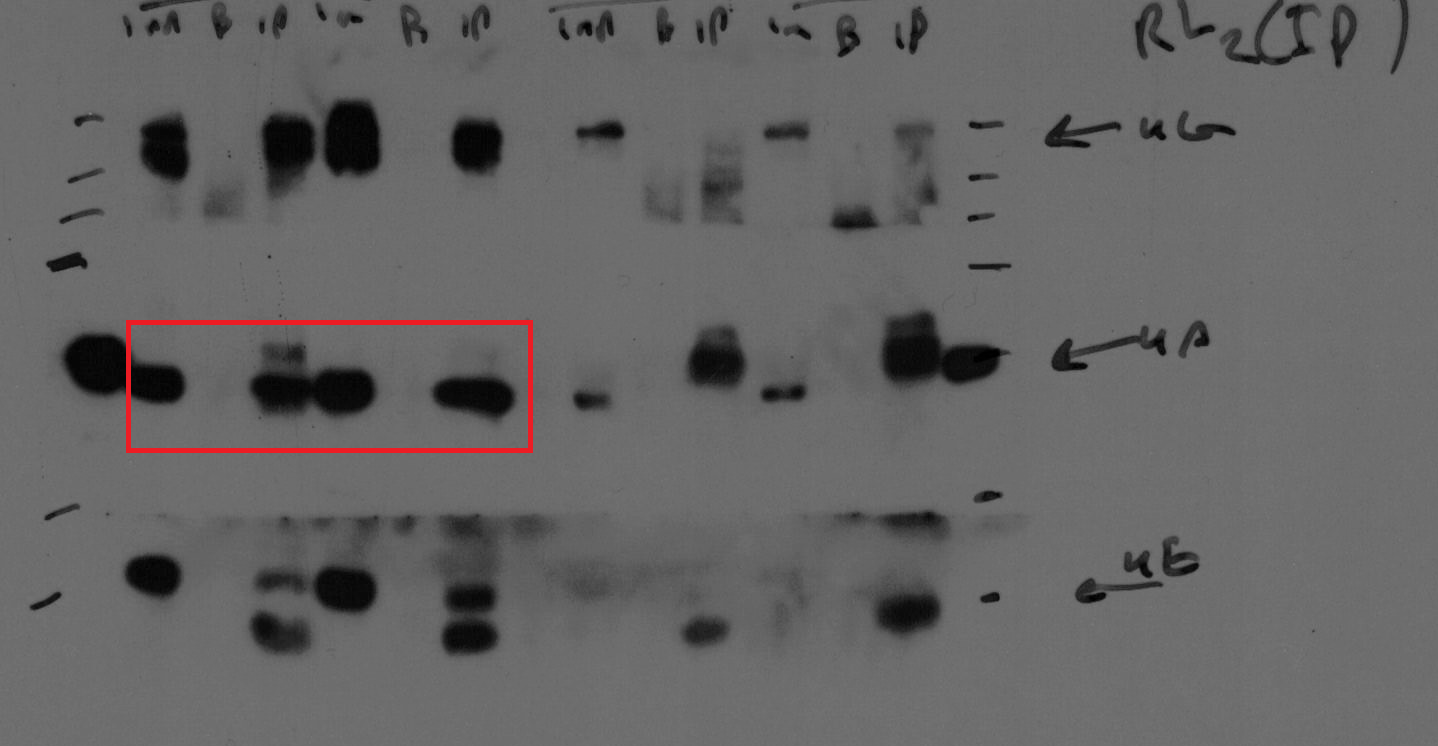

Supplement: Supplementary file 12 — Source Data [file 41467_2024_51678_MOESM12_ESM.zip › Source Data Zare et al/Fig. 8/Fig. 8d-Somata-eIF4A.tif]

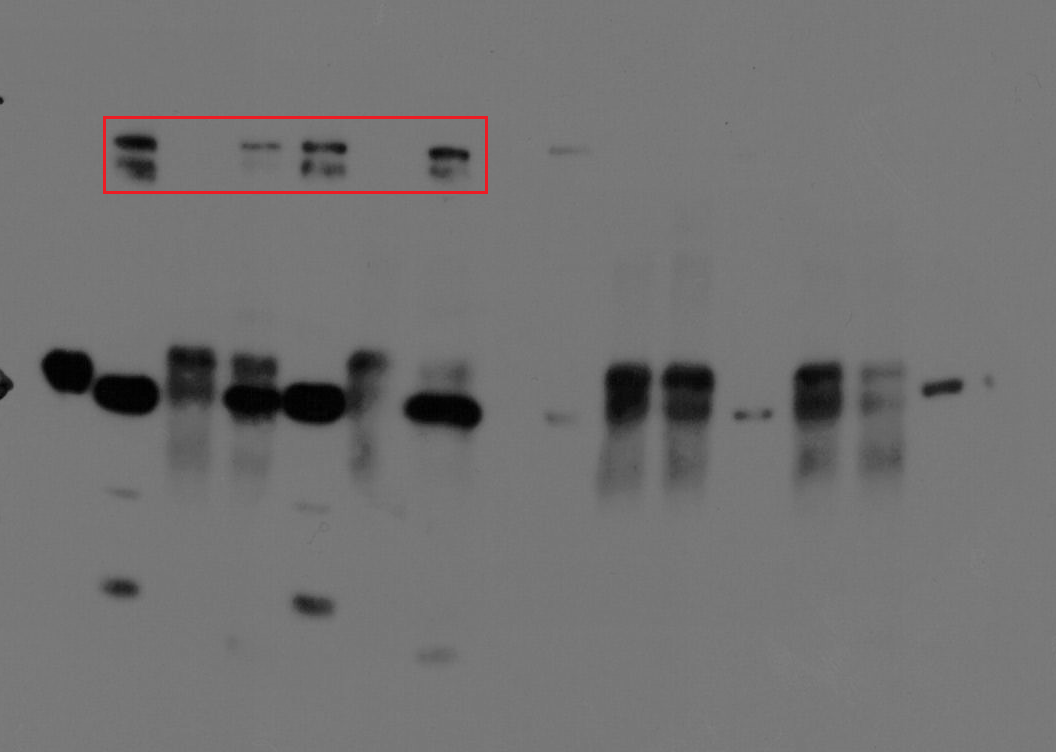

Supplement: Supplementary file 12 — Source Data [file 41467_2024_51678_MOESM12_ESM.zip › Source Data Zare et al/Fig. 8/Fig. 8d-Somata-eIF4G.tif]

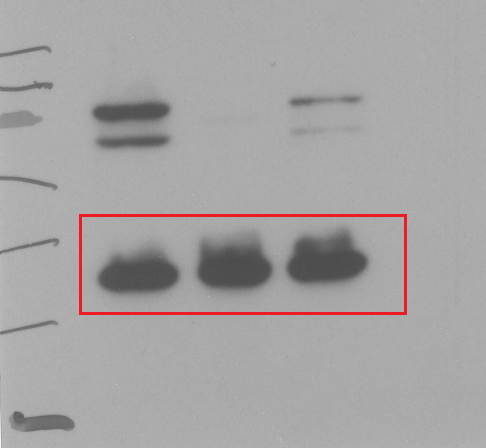

Supplement: Supplementary file 12 — Source Data [file 41467_2024_51678_MOESM12_ESM.zip › Source Data Zare et al/Supplementary Fig. 1/Supplementary Fig. 1d-Gapdh.tif]

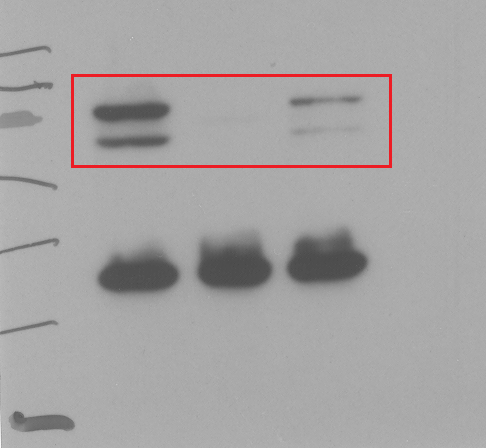

Supplement: Supplementary file 12 — Source Data [file 41467_2024_51678_MOESM12_ESM.zip › Source Data Zare et al/Supplementary Fig. 1/Supplementary Fig. 1d-hnRNP R.tif]

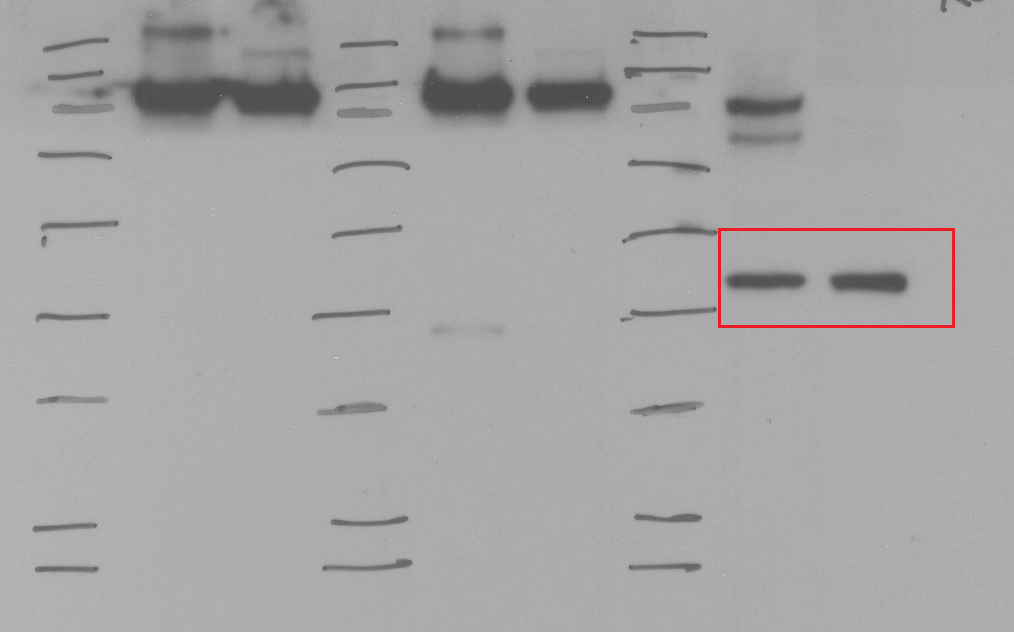

Supplement: Supplementary file 12 — Source Data [file 41467_2024_51678_MOESM12_ESM.zip › Source Data Zare et al/Supplementary Fig. 1/Supplementary Fig. 1f-Gapdh.tif]

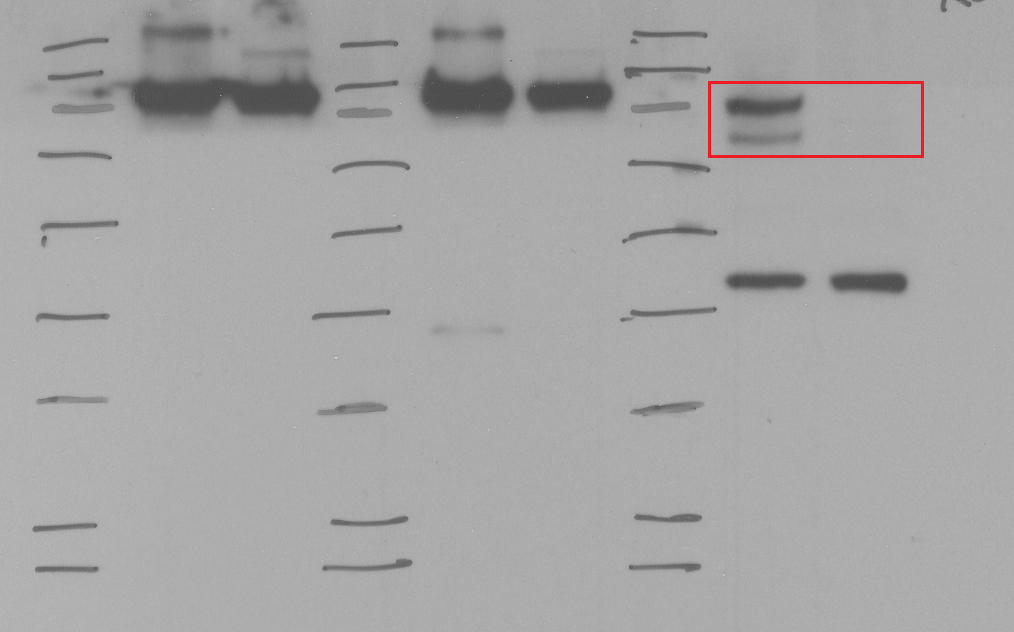

Supplement: Supplementary file 12 — Source Data [file 41467_2024_51678_MOESM12_ESM.zip › Source Data Zare et al/Supplementary Fig. 1/Supplementary Fig. 1f-hnRNP R.tif]

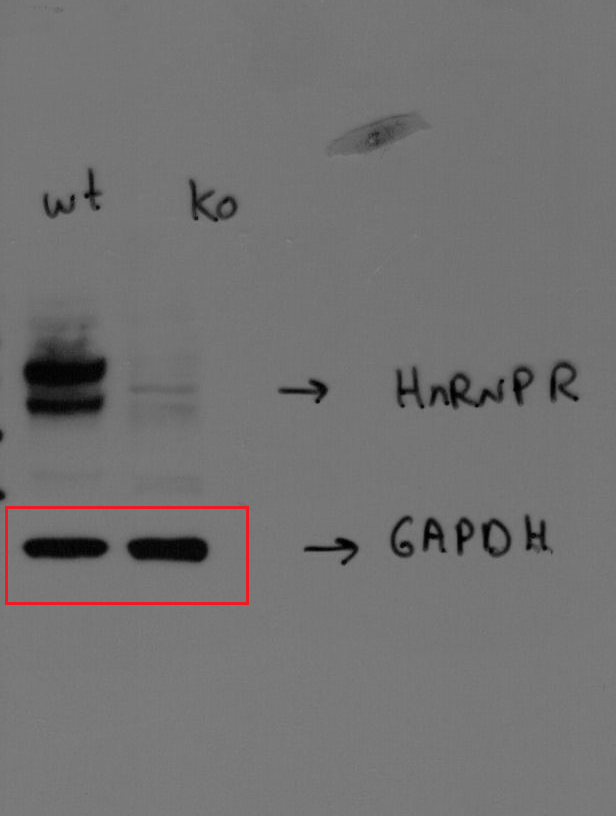

Supplement: Supplementary file 12 — Source Data [file 41467_2024_51678_MOESM12_ESM.zip › Source Data Zare et al/Supplementary Fig. 1/Supplementary Fig. 1g-Gapdh.tif]

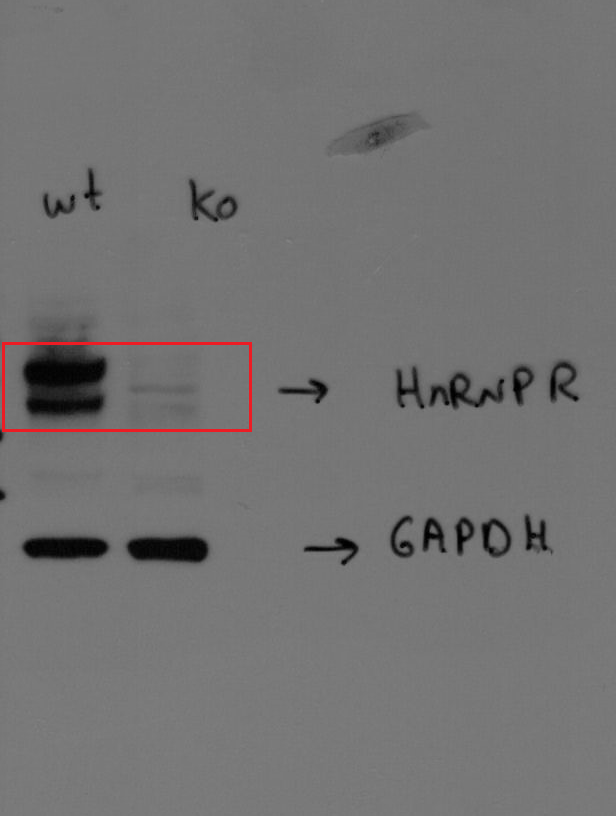

Supplement: Supplementary file 12 — Source Data [file 41467_2024_51678_MOESM12_ESM.zip › Source Data Zare et al/Supplementary Fig. 1/Supplementary Fig. 1g-hnRNP R.tif]

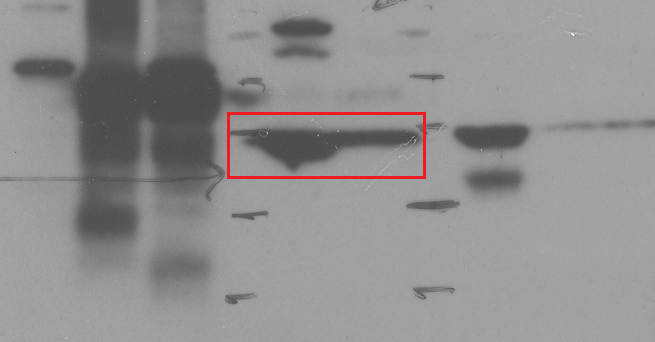

Supplement: Supplementary file 12 — Source Data [file 41467_2024_51678_MOESM12_ESM.zip › Source Data Zare et al/Supplementary Fig. 2/Supplementary Fig. 2b-Actb.tif]

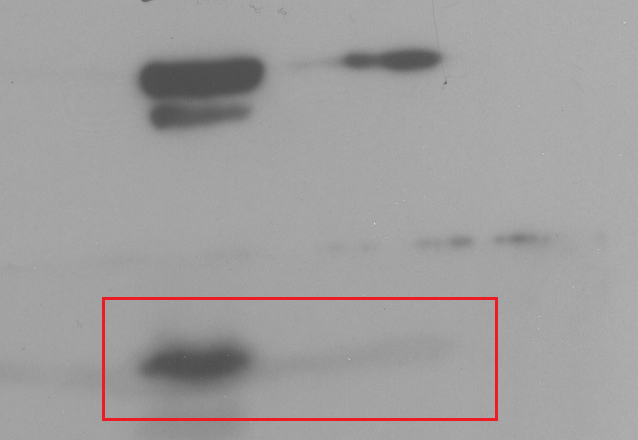

Supplement: Supplementary file 12 — Source Data [file 41467_2024_51678_MOESM12_ESM.zip › Source Data Zare et al/Supplementary Fig. 2/Supplementary Fig. 2b-Histon H3.tif]

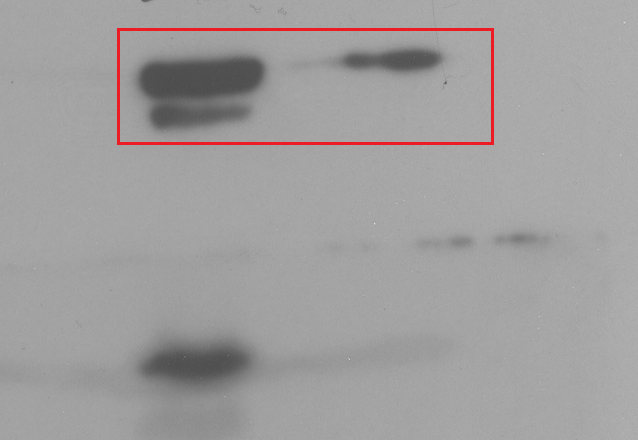

Supplement: Supplementary file 12 — Source Data [file 41467_2024_51678_MOESM12_ESM.zip › Source Data Zare et al/Supplementary Fig. 2/Supplementary Fig. 2b-Tubb3.tif]

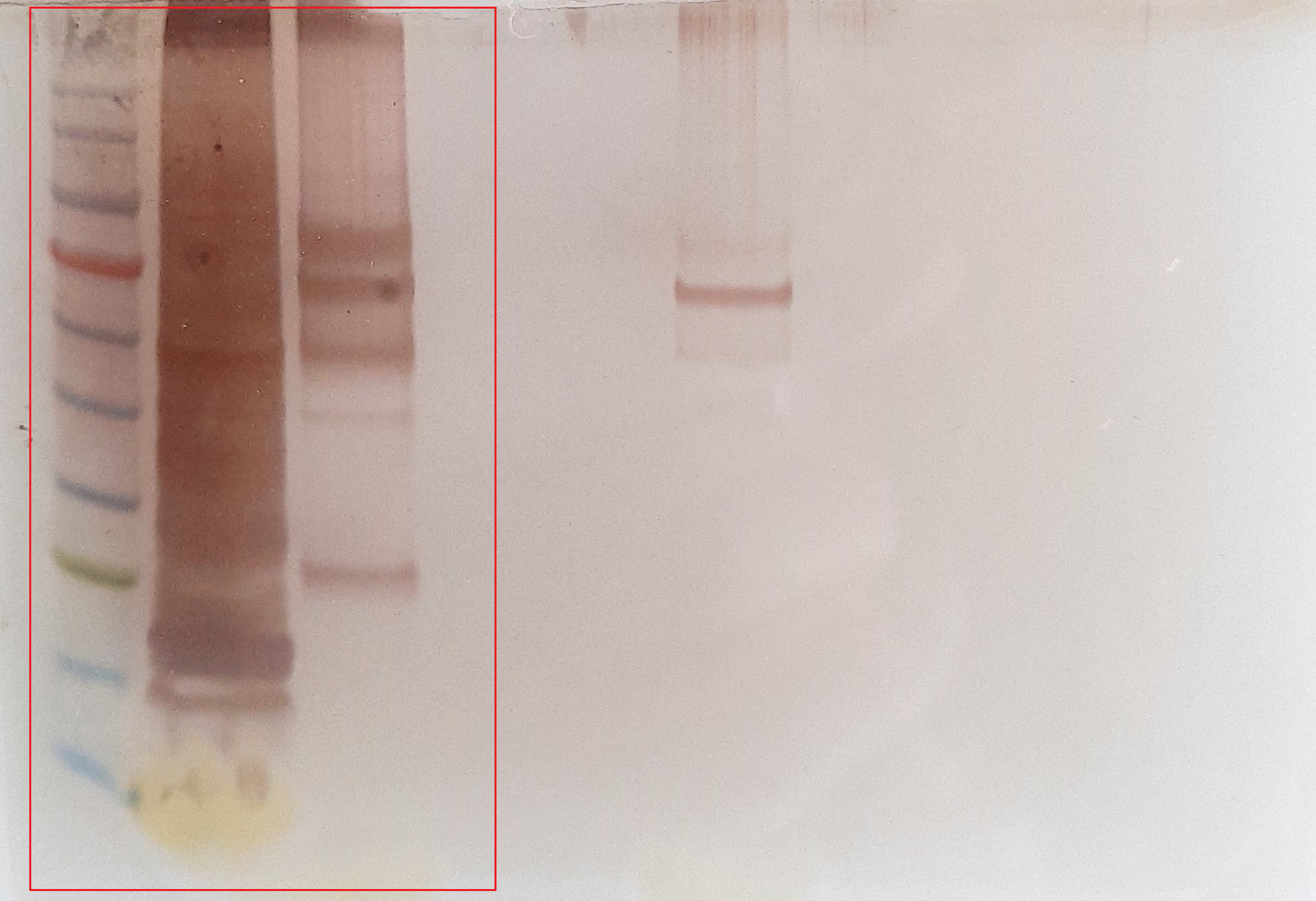

Supplement: Supplementary file 12 — Source Data [file 41467_2024_51678_MOESM12_ESM.zip › Source Data Zare et al/Supplementary Fig. 2/Supplementary Fig. 2C-long exposure.tif]

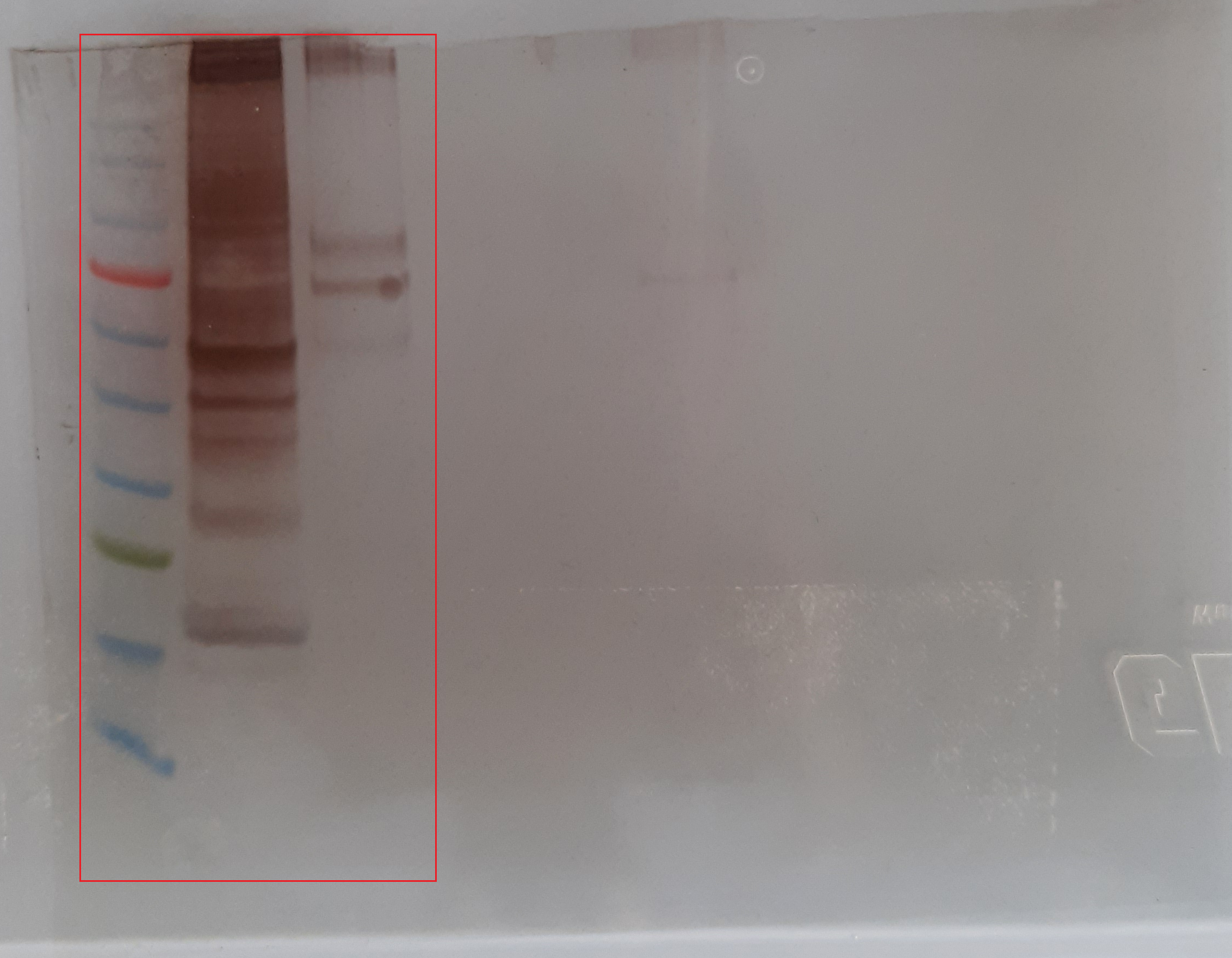

Supplement: Supplementary file 12 — Source Data [file 41467_2024_51678_MOESM12_ESM.zip › Source Data Zare et al/Supplementary Fig. 2/Supplementary Fig. 2C-short exposure.tif]
